# Vegetation transpiration contributions to precipitation recycling in a semi-arid grassland: an isotope-constrained source partitioning framework

**DOI:** 10.3389/fpls.2026.1867593

**Published:** 2026-07-30

**Authors:** Shaofeng Guo, Debin Jia, Mingyu Ji, Rong Wang, Donghui Zhang

**Affiliations:** 1 Water Conservancy and Civil Engineering College, Inner Mongolia Agricultural University, Hohhot, China; 2 Department of Water Conservancy and Civil Engineering, Hetao College, Bayannur, China; 3 Alshaa League Water Conservancy Engineering Technology Service Center, Alshaa Left Banner, China; 4 Institute of Remote Sensing Satellite, China Academy of Space Technology, Beijing, China

**Keywords:** land–atmosphere coupling, MODIS vegetation indices, plant-mediated precipitation recycling, semi-arid grassland, source partitioning, stable isotopes of precipitation, vegetation transpiration

## Abstract

**Introduction:**

Vegetation transpiration provides an important pathway through which semi‑arid grasslands return plant‑accessible water to the lower atmosphere, but its contribution to precipitation recycling remains difficult to quantify under sparse isotope observations and incomplete satellite records. Here, we developed an isotope‑constrained source partitioning framework to estimate the event‑ to annual‑scale contributions of vegetation transpiration, surface evaporation, and advected moisture in Zhenglan Banner, Inner Mongolia.

**Methods:**

The framework couples event‑scale precipitation isotopes with ERA5 and MODIS descriptors, explicitly encodes missing satellite predictors through value–mask pairs, and enforces a closed three‑endmember partition constrained by isotope consistency. Using 140 precipitation‑isotope events from 2018–2021 for model training and validation, the auxiliary isotope prediction achieved RMSE values of 2.62‰ for δ¹⁸O and 19.8‰ for δD, with near‑nominal 80% interval coverage of 0.83 and 0.82, respectively.

**Results:**

The trained framework was then used to reconstruct annual source‑fraction patterns during 2015–2024, including the vegetation‑transpiration fraction (f_tr_), surface‑evaporation fraction (f_ev_), and advected‑moisture fraction (f_adv_). The f_tr_ component formed coherent belts over vegetated areas and varied from 0.356 to 0.397, while f_adv_ ranged from 0.321 to 0.365 and f_ev_ remained lower and more spatially fragmented. Interannual variability was organized mainly along a recycling–advection axis: years with weaker large‑scale inflow showed expanded transpiration contribution, whereas stronger moisture convergence shifted the mixture toward advection. The retrieved f_tr_ was positively associated with independent vegetation and water‑flux indicators, including growing‑season NDVI, EVI, and MODIS ET_daily, providing indirect consistency with a vegetation‑mediated recycling signal rather than direct validation of transpiration‑derived precipitation recycling.

**Discussion:**

These results indicate that isotope‑constrained source partitioning can provide a data‑efficient way to quantify plant‑mediated precipitation recycling in semi‑arid grasslands. Future transfer of the framework should be accompanied by local checks of end‑member signatures and vegetation–atmosphere coupling conditions.

## 1 Introduction

Vegetation transpiration is a central pathway through which semi-arid grasslands return plant-accessible water to the lower atmosphere and participate in precipitation recycling. In water-limited ecosystems, vegetation is therefore not only a passive recipient of rainfall but also a mediator of land–atmosphere water exchange. Quantifying the contribution of transpiration-derived moisture to precipitation is important for diagnosing hydroclimatic sensitivity, ecosystem water availability, and grassland resilience under climate variability (Goursaud et al., 2018; Wang et al., 2020). The Inner Mongolia Autonomous Region (China) occupies a transition between the East Asian monsoon and the mid-latitude westerlies, a critical hydroclimatic boundary zone with strong interannual precipitation variability and tight land–atmosphere coupling across extensive grasslands and agro-pastoral mosaics (Yi and Alateng, 2014; Xu and Gou, 2016). In such settings, small shifts in the balance between large-scale advection and locally recycled vapor can alter rainfall efficiency, isotopic composition, and soil–plant water availability (Zhou and Li, 2018; Heydarizad et al., 2019). Separating vegetation transpiration from surface evaporation and externally advected moisture is therefore necessary for understanding how grassland vegetation contributes to the regional precipitation budget. Zhenglan Banner provides a useful testbed for this question because it is embedded in this regional hydroclimatic gradient and contains semi-arid vegetation, shallow groundwater influence, and event-scale isotope observations (Junjun et al., 2016; Sun et al., 2018). Conceptually, this framing builds on decades of work linking precipitation isotopes to moisture origin and phase-change history, from the meteoric-water line and condensation temperature effects to kinetic enrichment during evaporation and sub-cloud exchange (Yoshimura et al., 2003).

Recent syntheses show that continental precipitation often contains a substantial recycled component and that land-surface change can shift source regions and delivery pathways of moisture (Mingjun and Shengjie, 2016; Kong et al., 2023). For semi-arid grasslands, this recycled component has a direct plant-ecological meaning because it connects vegetation water use, canopy–atmosphere exchange, and the moisture supply contributing to precipitation. Eulerian–Lagrangian tracking and analytical frameworks have mapped the origin and fate of atmospheric water, emphasizing the dynamical controls on terrestrial recycling and its sensitivity to surface fluxes and circulation anomalies (YanWei et al., 2011; Fu et al., 2025). Over northern China, monsoon–westerlies interactions modulate moisture transport and vertical motion, yielding coherent swings in rainfall and extremes across the monsoon boundary zone, conditions emblematic of Inner Mongolia’s hydroclimate (Grande et al., 2024; Cai et al., 2025). These insights motivate a vegetation-aware view of precipitation recycling that is spatially explicit, event-resolved, and mechanistically interpretable, while still accounting for the competing roles of plant transpiration, surface evaporation, and advected vapor (Cui et al., 2025).

Stable water isotopes provide a practical basis for separating these moisture sources because they integrate moisture history across space and time (Szanto et al., 2007; Leroy-Dos Santos et al., 2020). Classical isotope theory explains how equilibrium and kinetic fractionation imprint δ^18^O, δD (equivalent to δ²H), and deuterium-excess on atmospheric vapor and precipitation as functions of condensation temperature, humidity, transport history, and re-evaporation, while global datasets such as GNIP provide empirical baselines that connect these fractionations to regional circulation regimes (Akers et al., 2020; Wei et al., 2023). However, using isotopes to identify vegetation-mediated recycling remains difficult in semi-arid interiors. *In-situ* isotope records are sparse, precipitation is intermittent, satellite variables are often incomplete during cloudy or convective periods, and two land-derived components—vegetation transpiration and open-water or soil evaporation—must be separated from externally advected moisture under limited event samples (Liu et al., 2024b; Zhang et al., 2025). These limitations restrict the direct use of isotope-informed mixing models for estimating plant-transpiration contributions at event and annual scales.

Here, we establish an isotope-constrained source partitioning framework designed to quantify vegetation transpiration contributions to precipitation recycling in a semi-arid grassland. First, we pair precipitation isotopes with reanalysis and satellite diagnostics while explicitly encoding missing predictors through value–mask pairs, allowing the framework to retain information from incomplete MODIS observations rather than discarding cloud-affected events (Holtz et al., 2025). Second, we adopt a small-sample learning strategy based on data-efficient tabular prior networks to stabilize inference under the event counts available in field isotope campaigns, avoiding the data demand of end-to-end deep models while retaining robustness through strong priors (Cheng et al., 2023; Lei et al., 2024). Third, we enforce a closed three-endmember partition of vegetation transpiration, surface evaporation, and advection (Masson-Delmotte et al., 2008; Hurley et al., 2012), together with isotope consistency informed by Craig–Gordon style flux fractionation. This design keeps the inferred fractions bounded and sum-to-one while linking them to interpretable drivers such as vegetation activity, surface energy balance, boundary-layer depth, vapor-pressure deficit, and moisture convergence.

At the application level, we adopt a regional-to-local framing. A well-documented circulation backdrop for eastern Inner Mongolia, where East Asian monsoon inflow interacts with mid-latitude westerlies and terrestrial recycling, anchors interpretation of the Zhenglan Banner maps and time series without presuming province-resolved estimates. All quantitative inference and mapping in this study are confined to Zhenglan Banner. Within that domain, we ask three linked questions: (1) can precipitation isotopes and satellite–reanalysis predictors separate the vegetation-transpiration fraction (f_tr_), surface-evaporation fraction (f_ev_), and advected-moisture fraction (f_adv_) under sparse event observations; (2) does the inferred f_tr_ carry an independent vegetation signal, as indicated by MODIS vegetation and evapotranspiration metrics; and (3) how does the balance between plant-mediated recycling and external advection vary across years in a semi-arid grassland? By addressing these questions, the study provides a spatially explicit and uncertainty-aware assessment of plant-mediated precipitation recycling that is relevant to grassland ecohydrology, vegetation–atmosphere coupling, and climate adaptation.

## 2 Materials and methods

### 2.1 The study area

**Figure 1:**
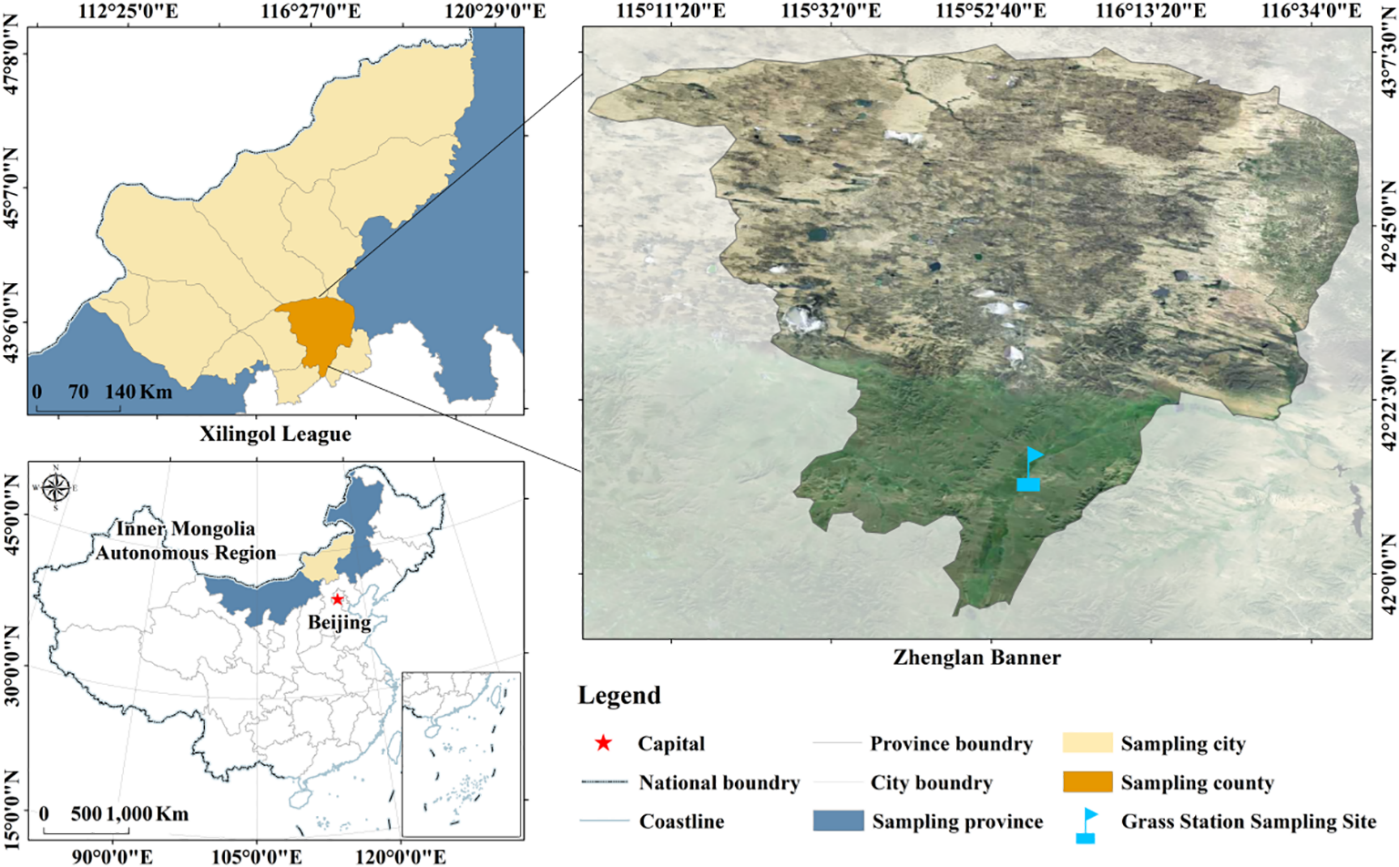
Location and sampling sites of the Zhenglan banner study area.

The study area is Zhenglan Banner, Xilin Gol League, Inner Mongolia, China (**Figure 1**) (Feng and Zhao, 2011; Qin et al., 2021). It occupies the central Eurasian temperate grassland belt and experiences a semi-arid continental climate with strong seasonality in both precipitation and temperature. Mean annual precipitation is about 350–400 mm and falls mainly from June to August; mean annual temperature is ~1–3 °C (Liu et al., 2022). The landscape comprises typical steppe, meadow steppe, sandy surfaces, and scattered water bodies underlain by a shallow groundwater system (Li et al., 2014). Vegetation is dominated by drought-tolerant grasses such as Leymus chinensis and Stipa grandis. Long-term observations from the Zhenglan Banner National Climate Observatory and nearby ecological stations provide high-quality meteorological and isotopic measurements that enable rigorous ground truth for satellite and reanalysis products.

Zhenglan Banner sits at the interface of the East Asian monsoon and mid-latitude westerlies, so moisture supply and boundary-layer structure vary systematically across seasons. This setting provides a useful ecological context for examining vegetation-mediated precipitation recycling because steppe vegetation, shallow groundwater influence, and strong seasonal water limitation jointly regulate plant water use and canopy–atmosphere exchange. Frequent convective episodes, pronounced sub-seasonal variability, and coexisting local and remote moisture inputs tend to imprint distinct signals on precipitation isotopes. These characteristics make the region well suited to separating vegetation transpiration, surface evaporation, and advected vapor, and to evaluating whether the inferred transpiration fraction carries an independent vegetation signal rather than only reflecting atmospheric transport.

### 2.2 Datasets

#### 2.2.1 Ground-based observations

**Figure 2:**
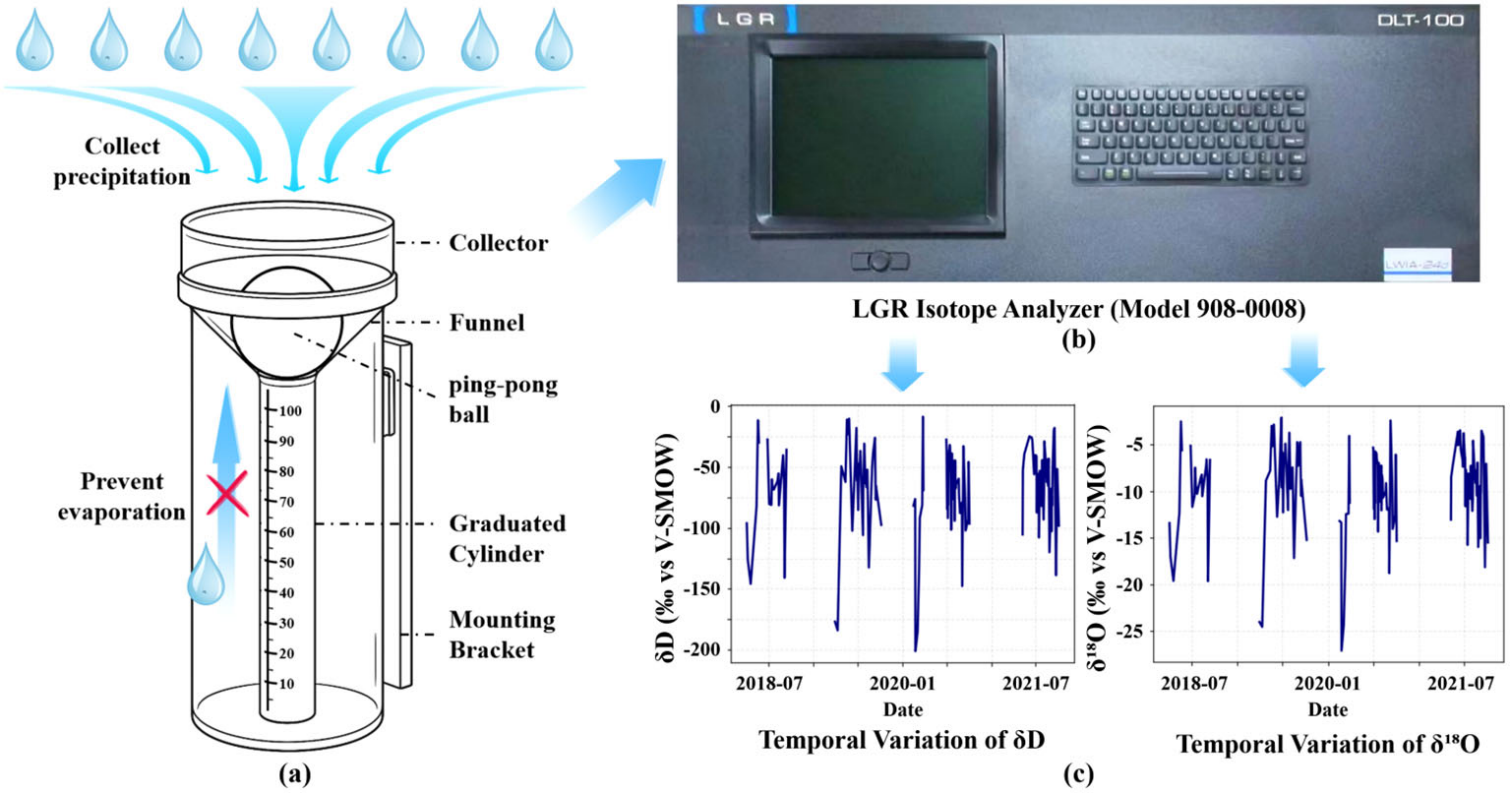
Precipitation sampling and isotope measurement workflow at the Zhenglan Banner Grass Station. **(A)** Schematic of the precipitation collector and anti-evaporation design. The funnel and graduated cylinder were used to collect event-scale precipitation, and the ping-pong ball was used to reduce evaporation during sampling. **(B)** Los Gatos Research (LGR) isotope analyzer, model 908-0008, used for δD and δ^18^O measurements. **(C)** Temporal variation of measured precipitation isotopes from 2018 to 2021, including δD and δ¹^8^O values relative to V-SMOW. This panel provides a visual check of the event-scale isotope sequence and complements the complete chronological records listed in **Table 1**.

Precipitation was sampled at the Zhenglan Banner Grass Station, Inner Mongolia, China (42°13′38″ N, 115°57′48″ E). The site is characterized by semi-fixed dunes with sandy to sandy-loam soils and natural shrublands dominated by Salix stands. From April 2018 to October 2021, we installed a custom collector (funnel plus polyethylene bottle) in an open, unobstructed area. A ping-pong ball was placed above the funnel to suppress evaporation during events. After each precipitation event, all samples were collected immediately, sealed, and transported on ice for freezer storage. In total, 140 event-scale precipitation samples were obtained. The sampling workflow is shown in **Figure 2**, and the full record of isotope measurements is listed in **Table 1**.

**Table 1:** Chronologically ordered precipitation δD and δ^18^O data from Zhenglan Banner (2018–2021).

Date	δD (‰)	δ^18^O (‰)	Date	δD (‰)	δ^18^O (‰)	Date	δD (‰)	δ^18^O (‰)
2018-4-1	-95.86	-13.37	2019-8-28	-51.02	-4.69	2020-9-27	-45.62	-6.03
2018-4-5	-125.58	-17.02	2019-9-1	-38.89	-7.22	2020-9-28	-51.78	-7.56
2018-4-17	-145.57	-19.58	2019-9-9	-25.79	-4.69	2020-10-1	-96.17	-15.3
2018-5-11	-82.18	-12.25	2019-9-12	-76.55	-10.45	2021-5-7	-105.09	-13.01
2018-5-17	-11.39	-2.47	2019-9-13	-65.21	-8.71	2021-5-8	-52.39	-8.45
2018-5-21	-29.69	-5.57	2019-10-5	-97.26	-15.19	2021-5-14	-39.09	-7.28
2018-6-25	-27.22	-5.08	2020-2-14	-81.46	-13.15	2021-6-4	-24.58	-3.57
2018-7-11	-80.82	-10.45	2020-2-21	-76.03	-13.33	2021-6-5	-25.12	-5.03
2018-7-13	-59.85	-7.43	2020-2-22	-200.76	-27.06	2021-6-13	-25.42	-3.42
2018-7-16	-66.61	-9.29	2020-3-2	-184.86	-24.28	2021-6-16	-28.31	-5.55
2018-7-20	-80.12	-11.64	2020-3-8	-134.23	-17.89	2021-6-23	-55.73	-6.64
2018-7-21	-68.3	-10.24	2020-3-11	-91.41	-12.41	2021-6-24	-54.29	-7.34
2018-8-5	-61.22	-8.47	2020-3-21	-81.02	-12.37	2021-6-27	-39.93	-3.74
2018-8-6	-59.84	-8.44	2020-3-24	-8.49	-4.03	2021-7-2	-40.43	-6.31
2018-8-9	-55.02	-8.18	2020-3-26	-68.65	-11.18	2021-7-3	-41.08	-6.33
2018-8-11	-81.24	-10.5	2020-6-28	-27.29	-5.28	2021-7-4	-87.05	-11.58
2018-8-27	-42.28	-7.1	2020-6-29	-36.7	-5.38	2021-7-5	-56.35	-7.64
2018-8-28	-40.08	-6.54	2020-7-1	-84.28	-11.88	2021-7-6	-55.04	-7.66
2018-9-1	-61.18	-8.51	2020-7-2	-82.41	-11.3	2021-7-12	-69.37	-8.77
2018-9-11	-35.6	-6.6	2020-7-3	-68.01	-7.81	2021-7-13	-107.58	-15.72
2018-9-30	-140.61	-19.61	2020-7-4	-91.6	-12.95	2021-7-17	-52.86	-8.07
2019-3-29	-176.54	-23.99	2020-7-5	-75.28	-8.74	2021-7-18	-64.63	-9.31
2019-4-9	-183.72	-24.51	2020-7-6	-80.79	-11.26	2021-7-24	-81.69	-10.76
2019-4-24	-49.14	-8.82	2020-7-7	-40.63	-5.69	2021-7-25	-73.89	-10.77
2019-5-11	-62.02	-7.76	2020-7-12	-31.69	-5.92	2021-7-26	-44.07	-7.22
2019-5-17	-10.9	-2.95	2020-7-13	-32.11	-6.51	2021-8-1	-92.81	-12.07
2019-5-18	-13.78	-3.38	2020-7-17	-56	-7.13	2021-8-3	-68.78	-10.81
2019-5-19	-22.98	-5.17	2020-7-18	-101.24	-14.29	2021-8-4	-28.69	-4.54
2019-5-26	-10.11	-2.81	2020-7-21	-38.64	-6.92	2021-8-9	-42.57	-7.26
2019-6-8	-102.04	-12.7	2020-7-27	-69.87	-10.37	2021-8-14	-62.14	-9.06
2019-6-16	-62.93	-8.93	2020-7-28	-76.25	-11.09	2021-8-15	-59.12	-8.73
2019-6-25	-17.75	-2.06	2020-7-31	-44.51	-7.2	2021-8-16	-43.71	-7.77
2019-6-26	-37.54	-6.37	2020-8-1	-93.89	-12.02	2021-8-19	-64.96	-10.42
2019-6-27	-60.74	-9.57	2020-8-3	-44.28	-8.23	2021-8-22	-42.83	-6.88
2019-6-29	-56.88	-5.78	2020-8-5	-67.42	-8.78	2021-8-23	-69.03	-10.32
2019-7-2	-84.99	-12.23	2020-8-7	-63.78	-10.65	2021-8-25	-119.69	-15.94
2019-7-5	-47.59	-6.28	2020-8-18	-59.29	-9.96	2021-9-2	-67.38	-9.38
2019-7-9	-45.42	-6.74	2020-8-19	-72.07	-9.09	2021-9-3	-102.45	-15
2019-7-11	-36.57	-4.86	2020-8-23	-87.45	-11.93	2021-9-4	-73.74	-10.34
2019-7-22	-105.62	-11.98	2020-8-24	-87.55	-11.73	2021-9-5	-81.58	-9.94
2019-7-23	-69.09	-8.39	2020-8-31	-78.85	-12.06	2021-9-7	-50.33	-3.47
2019-7-27	-30.6	-3.68	2020-9-1	-147.32	-18.75	2021-9-12	-19.41	-3.88
2019-8-1	-63.19	-9.71	2020-9-4	-77.21	-10.25	2021-9-15	-17.66	-4.18
2019-8-5	-59.79	-8.74	2020-9-6	-32.64	-2.37	2021-9-21	-138.51	-18.11
2019-8-6	-66.37	-8.63	2020-9-13	-75.52	-9.01	2021-9-26	-51.41	-7.01
2019-8-9	-51.95	-7.4	2020-9-15	-101.88	-14.01	2021-10-3	-97.85	-15.47
2019-8-15	-132.17	-17.14	2020-9-25	-92.87	-12.54			

The precipitation isotope records are arranged chronologically by sampling date. The multi-column layout is used only for compact presentation and does not indicate separate sampling groups.

Hydrogen and oxygen isotopes were analyzed with a Los Gatos Research (LGR) liquid water isotope analyzer, model 908–0008 at Inner Mongolia Agricultural University (Kurita et al., 2012; Emanuelsson et al., 2015). Each sample was injected six times; the first two injections were discarded and the mean of the remaining four was used as the final value. Routine laboratory QA/QC, including calibration with standards, drift correction, memory control, and inspection of injection stability, ensured accuracy and comparability. Potential analytical outliers were checked during this QA/QC procedure, but no additional statistical outlier deletion was applied to the raw isotope record. Results for δ18O and δD are reported as per-mil deviations relative to V-SMOW (Johnson et al., 2011). The winsorization described in the predictor-preprocessing section was applied only to ERA5/MODIS event-window predictor statistics before model fitting, not to the raw precipitation isotope measurements. Therefore, the values reported in **Table 1** represent laboratory quality-controlled isotope measurements after routine processing, rather than winsorized values.

#### 2.2.2 Satellite and reanalysis predictors of vegetation–atmosphere coupling

Satellite and reanalysis variables were used to characterize the atmospheric transport, vegetation condition, surface energy balance, and hydrometeorological setting that regulate the vegetation-transpiration fraction (f_tr_), surface-evaporation fraction (f_ev_), and advected-moisture fraction (f_adv_). Ground-based precipitation isotopes served only as training targets and validation data, whereas ERA5 and MODIS predictors provided scalable environmental descriptors for source-fraction estimation. This design allows the inferred f_tr_ to be interpreted against independent vegetation and water-flux indicators while avoiding reliance on additional isotope observations outside 2018–2021. No additional isotope data were used for 2015–2017 or 2022–2024; maps for these periods are therefore out-of-time inferences.

ERA5 predictors describe the atmospheric transport and boundary-layer setting within which plant-mediated recycling occurs, including boundary-layer depth, integrated water vapor, near-surface temperature and humidity, wind fields, vertical velocity, and surface radiation. These variables constrain mixing depth, convective initiation, surface evaporative demand, and long-range moisture transport (**Table 2**) (He et al., 2023; Lang et al., 2023; Wang et al., 2023).

**Table 2:** ERA5 atmospheric variables used to characterize transport, boundary-layer state, and moisture supply in the source-partitioning framework.

Abbrev.	Full name (ERA5)	Description/units	Core relevance
BLH	Boundary Layer Height	Planetary boundary-layer height (m)	Indicates mixing depth and local moisture recycling potential; constrains convective development and its influence on δD and δ^18^O enrichment or depletion.
E	Evaporation	Total surface evaporation (m of water; ERA5 sign convention is negative for upward flux, we use −E as magnitude)	Provides the surface evaporation flux driving the f_ev_ end-member and associated isotope enrichment.
SSR	Surface Solar Radiation Downwards	Downward short-wave radiation (J m^-^²; integrated over the event window)	Key control on surface energy balance and evapotranspiration strength, informing vegetation transpiration (f_tr_) and surface evaporation (f_ev_) (Jiang et al., 2020).
T2m	2-Metre Temperature	Near-surface air temperature (°C; converted from K)	Governs equilibrium and kinetic fractionation coefficients; constrains temperature dependence of δD and δ^18^O and local recycling intensity (Munoz-Sabater et al., 2021).
TCWV	Total Column Water Vapor	Integrated column water vapor (kg m^-^²)	Describes total atmospheric moisture and large-scale transport background, critical for estimating advected moisture fraction f_adv_ (Xu and Liu, 2023).
TP	Total Precipitation	Accumulated precipitation (m)	Direct measure of event-scale rainfall amount; used for normalizing end-member contributions and validating isotope predictions.
Td2m	2-Metre Dew-Point Temperature	Dew-point temperature at 2 m (°C; converted from K)	Represents near-surface relative humidity, constraining evaporative fractionation and δ–RH relationships, essential for f_ev_ estimation.
U10	10-Metre U-Component of Wind	East–west wind speed at 10 m (m s^-^¹)	With V10, defines horizontal moisture transport pathways supporting the advected moisture end-member f_adv_ (Stante et al., 2023).
V10	10-Metre V-Component of Wind	North–south wind speed at 10 m (m s^-^¹)	Complements U10 to resolve wind vectors and diagnose external moisture inflow.
omega500	Vertical Velocity at 500 hPa	Mid-tropospheric vertical velocity (Pa s^-^¹)	Captures large-scale ascent or subsidence, identifying convective triggers and synoptic controls on isotope variability.
q850	Specific Humidity at 850 hPa	Mid-tropospheric specific humidity (kg kg^-^¹)	Represents lower-tropospheric moisture content; key for distinguishing remote moisture transport from local recycling and quantifying f_adv_.

MODIS products were used to represent vegetation condition and surface-energy controls on plant-mediated recycling, including albedo, NDVI, EVI, daily evapotranspiration, and daytime/nighttime land-surface temperature (**Table 3**). NDVI and EVI characterize canopy greenness and phenological state, ET_daily represents total evapotranspiration, including both plant transpiration and surface/soil evaporation, and LST variables describe surface heating and evaporative demand. Data completeness varies across products: Albedo, NDVI, and EVI are nearly complete, ET_daily is missing only 3.6% of samples, whereas LST_day and LST_night show ~70–75% gaps due to frequent cloud cover (**Table 4**). Instead of discarding these variables, we preserve the missingness pattern as an informative signal of cloudy and convective conditions that can influence isotope enrichment, surface energy balance, and vegetation–atmosphere coupling (Lu et al., 2023; Yilmaz, 2023).

**Table 3:** MODIS vegetation and surface-energy predictors used to constrain plant-mediated recycling and surface evaporation.

Abbrev.	Full name (MODIS product)	Description/units	Core relevance
Albedo	Surface Albedo (MCD43 series)	Broadband shortwave surface reflectance/albedo, dimensionless (0–1)	Controls surface energy balance and net radiation, informing evapotranspiration intensity and its influence on δD and δ^18^O through vegetation and soil moisture feedbacks (Pang et al., 2025).
ET_daily	Daily Evapotranspiration (MOD16)	Daily total evapotranspiration (mm day^-^¹)	Direct measure of combined soil evaporation and plant transpiration, a primary predictor of the recycled moisture fraction f_tr_ and f_ev_.
EVI	Enhanced Vegetation Index (MOD13)	Spectral vegetation index (dimensionless)	Sensitive to canopy structure and chlorophyll; constrains vegetation phenology and transpiration strength affecting f_tr_ and isotopic enrichment.
LST_day	Land Surface Temperature (Daytime) (MOD11)	Skin temperature of the land surface in daytime (K or °C)	Indicates daytime surface heating and potential evaporative demand, key for diurnal fractionation processes and local recycling signals.
LST_night	Land Surface Temperature (Nighttime) (MOD11)	Skin temperature of the land surface at night (K or °C)	Captures nighttime cooling and boundary-layer stability, complementing LST_day for assessing daily energy balance and dew/fog formation impacts on isotopes.
NDVI	Normalized Difference Vegetation Index (MOD13)	Spectral vegetation index (dimensionless)	Provides a long-term measure of vegetation greenness and biomass, supporting estimation of phenological transpiration (f_tr_) and canopy–atmosphere moisture exchange (Zandler et al., 2020).

**Table 4:** Coverage and missing-value statistics of MODIS vegetation and surface-energy predictors for 140 precipitation events.

Variable	Missing count	% Missing (out of 140)
Albedo	0	0%
ET_daily	5	3.60%
EVI	0	0%
LST_day	104	74.30%
LST_night	99	70.70%
NDVI	0	0%

To improve reproducibility, we added a dedicated preprocessing workflow for the ERA5 and MODIS predictors. ERA5 atmospheric variables were obtained from the Copernicus Climate Data Store, including hourly single-level and pressure-level reanalysis fields on a 0.25° × 0.25° regular latitude–longitude grid (https://cds.climate.copernicus.eu/). MODIS predictors were obtained from NASA Earthdata and LP DAAC, including MCD43A3 albedo at 500 m and daily temporal resolution, MOD13A1 NDVI/EVI at 500 m and 16-day temporal resolution, MOD16A2 evapotranspiration at 500 m and 8-day temporal resolution, and MOD11A1 daytime/nighttime land-surface temperature at 1 km and daily temporal resolution (https://search.earthdata.nasa.gov/; https://lpdaac.usgs.gov/).

All predictors were converted to a common event-level representation before model construction. For each precipitation event, the event window Ωt was defined as a 72-h window centered on the recorded precipitation event time, spanning 36 h before to 36 h after the event center. When the exact start and end times of a precipitation event were available and the event lasted longer than 72 h, the window was expanded to cover the full observed event duration. For event records without detailed start–end timing, the sampling date was used as the event date, and the 72-h centered window was applied consistently.

Spatial harmonization was performed using a 25-km radius buffer centered on the precipitation sampling site for event-scale model training. For gridded spatial prediction, the same 25-km radius buffer was centered on each target grid cell. ERA5 variables were bilinearly sampled or spatially averaged over the buffer depending on grid-cell coverage, whereas MODIS variables were quality-screened and averaged over all valid pixels within the same buffer. This design avoided forcing all products to an artificial common pixel size and instead converted multi-resolution inputs into consistent event-level statistics.

Temporal matching was product-specific. ERA5 hourly variables were extracted over Ωt and summarized using fixed statistics, including mean, quantiles, extrema, duration-related descriptors, and linear trend. MODIS daily products, including MCD43A3 and MOD11A1, were matched to the event date or the nearest valid observation within Ωt. MOD16A2 8-day evapotranspiration and MOD13A1 16-day NDVI/EVI were assigned according to the composite period covering the event center. If no valid MODIS pixel was available within the spatial buffer and temporal matching window, the value was set to the robust center after standardization and the corresponding missingness mask was set to 1.

After temporal matching and spatial aggregation, all predictor statistics were winsorized at the 1st and 99th percentiles using training-fold statistics only, and then robustly standardized using the training-fold median and median absolute deviation. The same transformation parameters were applied unchanged to validation and test folds to avoid temporal leakage. The resulting value channel was concatenated with a binary missingness channel. Together, these procedures provide a reproducible link between multi-resolution ERA5/MODIS data and the event-scale isotope-constrained source-partitioning framework.

### 2.3 Isotope-constrained framework for estimating vegetation transpiration contributions

The source-partitioning framework was designed to estimate the vegetation-transpiration contribution to precipitation recycling while retaining physical closure among the three moisture sources. The framework uses precipitation isotopes as event-scale constraints and combines them with ERA5 and MODIS predictors that describe atmospheric transport, vegetation condition, surface energy balance, and satellite-data availability. The model returns two linked outputs: probabilistic isotope predictions used to evaluate reliability, and simplex-constrained source fractions, including f_tr_, f_ev_, and f_adv_.

#### 2.3.1 Event-window predictor construction and missingness encoding

**Figure 3:**
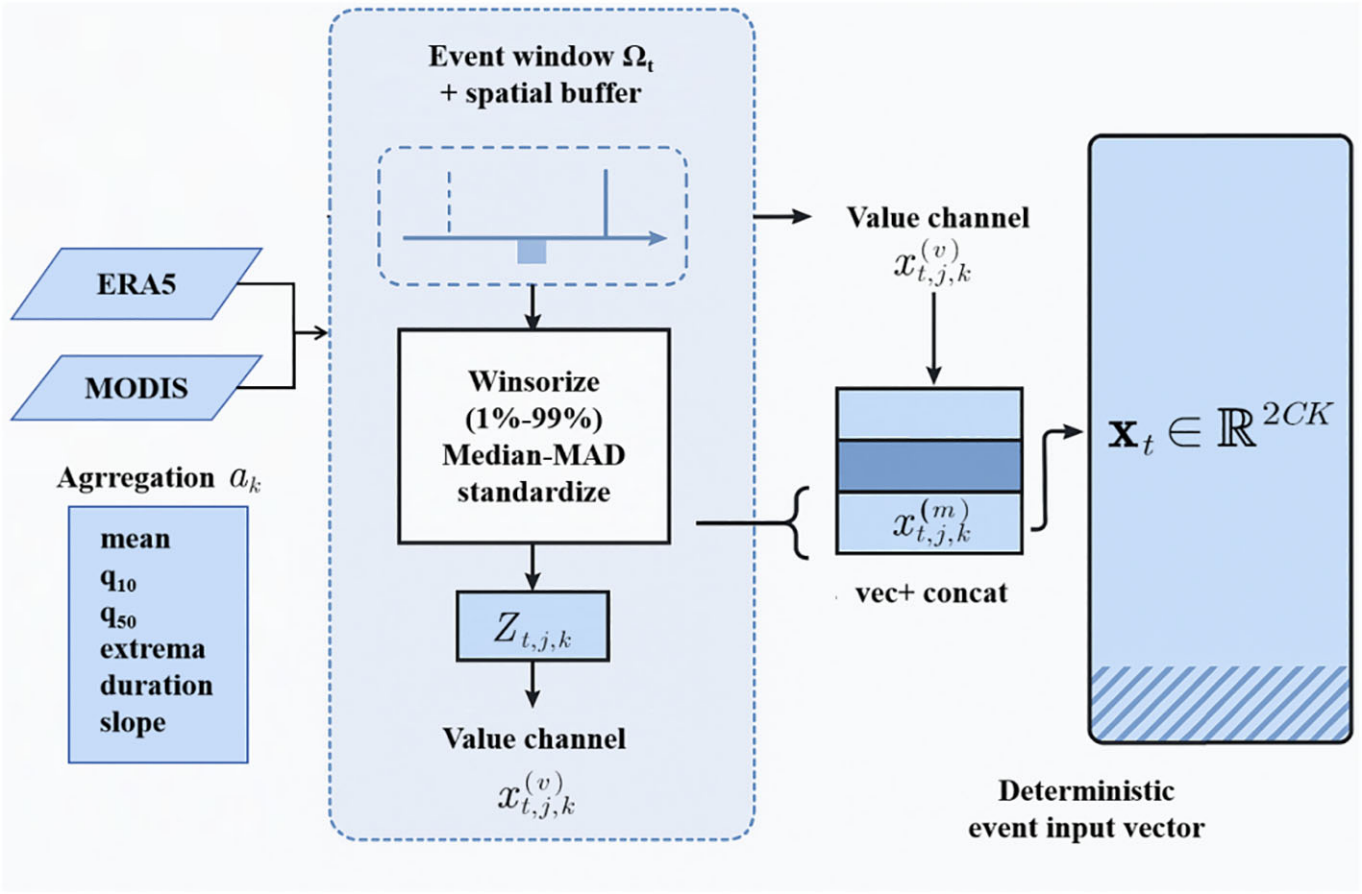
Event-scale multi-source preprocessing and missingness encoding workflow. .

Estimating vegetation-transpiration contributions from event-scale precipitation requires predictors with different temporal cadences, spatial supports, and missing-data patterns to be represented consistently. ERA5 and MODIS inputs are therefore cast into an event-centric representation: aligned within a common event window, summarized by stable statistics, and standardized onto a commensurate scale (**Figure 3**) (Zhong et al., 2020; Chen et al., 2025). Missingness is treated explicitly as signal rather than filled through unconstrained imputation: absent values default to a robust center, while a binary mask preserves the missing pattern. This design is important for vegetation–atmosphere applications because cloud-related MODIS gaps may coincide with convective conditions, surface-energy changes, and isotope shifts. All transforms are fixed from training periods and applied unchanged thereafter to avoid temporal leakage (Feng et al., 2023; Gunasekara, 2025). The result is a deterministic and reproducible input vector that links event-scale isotope information with vegetation, surface-energy, and atmospheric predictors.

(a) Unified Event-Window Aggregation and Robust Scaling

For each precipitation event t, ERA5/MODIS variables xj are temporally aligned within the event window Ωt defined above, namely a 72-h window centered on the recorded event time and expanded to the full observed duration for longer events when detailed timing is available (Liu et al., 2024a; Yu et al., 2025a). All variables are sampled within the same 25-km radius spatial buffer centered on the sampling site or target grid cell. Each variable is summarized with a fixed family of operators, including mean, quantiles q10/q50/q90, extrema, valid-observation count, missing-data fraction, and linear trend, yielding per-event statistics (Kilian, 1998).

(1) $${\text{s}}_{\text{t,j,k}}={\text{a}}_{\text{k}}\left({\text{v}}_{\text{j}}(\tau ):\tau \in \Omega_{\text{t}}\right),\text{ k}=1,\ldots ,\text{K}$$

where j indexes source variables/sensors and a_k_ denotes the aggregation operator. This produces a harmonized description of heterogeneous signals at the event scale.

To control outliers and reconcile heterogeneous units under small-n conditions, each statistic is winsorized at the 1st and 99th percentiles (estimated on training folds only) and then robustly standardized using median–MAD:

(2) $${\tilde{s}}_{\text{t,j,k}}=\text{clip}\left({\text{s}}_{\text{t,j,k}},{\text{q}}_{\text{j,k}}^{0.01}{\text{,q}}_{\text{j,k}}^{0.99}\right),\;{\text{z}}_{\text{t,j,k}}=\frac{{\tilde{s}}_{\text{t,j,k}}-{\text{med}}_{\text{j,k}}}{{\text{MAD}}_{\text{j,k}}+\epsilon }$$

Here ${\text{q}}_{\text{j,k}}^{\text{p}}$, med_j,k_ and MAD_j,k_ are computed exclusively on the training data and applied unchanged to validation/test to prevent temporal leakage; ϵ is a small constant for numerical stability. This transformation yields dimensionally consistent, outlier-resistant features suitable for downstream learning.

All normalized entries {z_t,j,k_} are stacked in a fixed, deterministic order (by variable j then statistic k) into a single vector ${\text{z}}_{t}\text{= vec}\left({\text{z}}_{\text{t,j,k}}\right)\in {\text{R}}^{\text{CK}}$, serving as the value channel; the final model input concatenates a binary missingness channel.

(b) Explicit missingness encoding and data partitioning

Missing data are treated explicitly, not imputed (Guan et al., 2024). After robust scaling, let z_t,j,k_ denote the standardized statistic for event t, variable j, and operator k. A binary mask ${\text{m}}_{\text{t,j,k}}\in \{0,1\}$ (1 = missing within the event window; 0 = observed) supports a two-channel representation:

Value channel — observed entries retained, missing entries set to the robust center:

(3) $${\text{x}}_{\text{t,j,k}}^{\left(\text{v}\right)}=\left(1-{\text{m}}_{\text{t,j,k}}\right){\text{z}}_{\text{t,j,k}}.$$

Missingness channel — the absence pattern recorded directly:

(4) $${\text{x}}_{\text{t,j,k}}^{\left(\text{m}\right)}={\text{m}}_{\text{t,j,k}}$$

Both channels are stacked in a fixed order to form the model input,

(5) $${\text{x}}_{t}=\left[\text{vec}\left({\text{x}}_{\text{t,}1:\text{C,}1:\text{K}}^{\left(\text{v}\right)}\right);\text{vec}\left({\text{x}}_{\text{t,}1:\text{C,}1:\text{K}}^{\left(\text{m}\right)}\right)\right]\in {\text{R}}^{2\text{CK}}$$

yielding an interpretable, numerically stable vector in which zeros align with the robust center. This design allows the model to exploit systematic gap structure.

For partitioning, a time-aware split (leave-one-year-out) is adopted (Barreda et al., 2024; Ghosh et al., 2024). All winsorization bounds, medians, and MADs are estimated only on training folds and applied unchanged to validation/test to prevent temporal leakage; feature ordering is fixed across splits to ensure determinism and reproducibility.

#### 2.3.2 Small-sample backbone and output heads

A prior-data-fitted network (PFN) is a model pre-trained on synthetic tabular tasks so that it can perform data-efficient inference on small tabular datasets. In this study, we used the second version of the tabular PFN model (TabPFN-v2) to stabilize source-fraction estimation under sparse isotope samples, heterogeneous predictors, and structured MODIS gaps (Rundel et al., 2024; Lin et al., 2025). The encoder directly ingests the standardized event vector with robustly scaled statistics and an explicit missingness channel, and yields a low-variance event embedding suited to isotope–environment relations. From this shared embedding, one head produces distributional estimates for δ^18^O and δD at prescribed quantiles (Jareno et al., 2020), and another returns simplex-constrained fractions for f_tr_, f_ev_, and f_adv_. The design preserves parsimony and interpretability while anchoring the inferred ftr to isotope consistency and three-endmember closure (Khanmohammadi et al., 2024; Ruiz-Villafranca et al., 2025).

**Figure 4:**
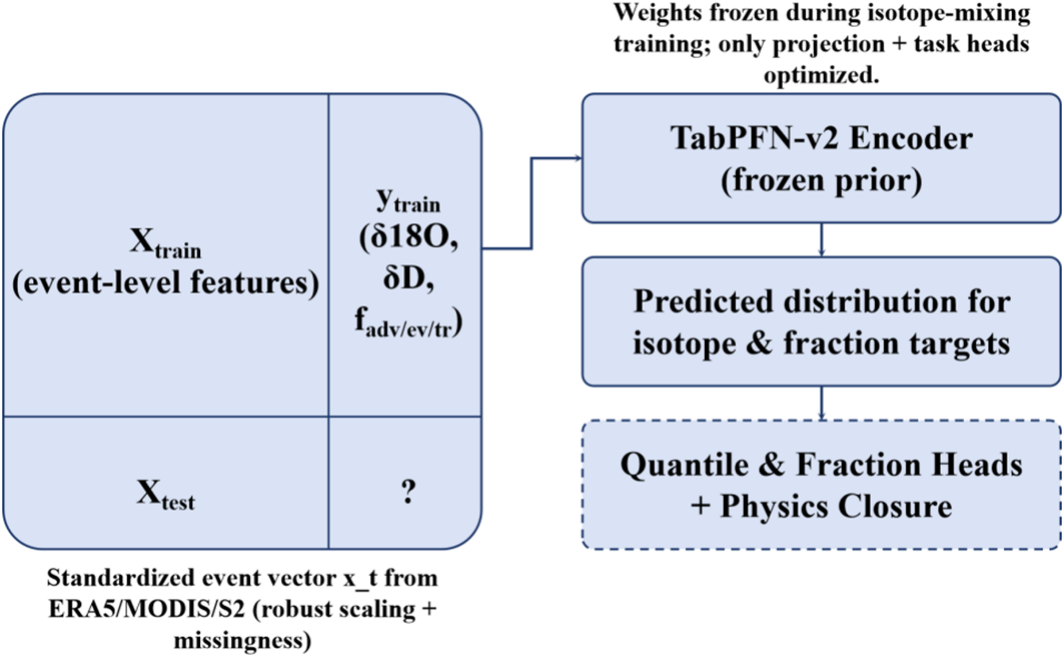
TabPFN-v2 pretraining and event-scale inference workflow. TabPFN-v2 is pre-trained on synthetic tabular datasets to learn a prior Θ^*^ that performs approximate Bayesian inference in a single forward pass. In deployment, the standardized event vector ${\text{x}}_{t}\in {\text{R}}^{2\text{CK}}$ is encoded by the frozen PFN backbone to produce a shared embedding e_t_, which feeds a quantile-regression head for δD and δ^18^O and a simplex-constrained component-fraction head (f_tr_, f_ev_, f_adv_). Outputs are coupled by a three-component mixing closure, and training optimizes only the projection and heads while keeping Θ^*^ fixed.

The overall data flow—from the standardized event vector x_t_ to the frozen PFN backbone, the two task heads, and the physics closure—is summarized in **Figure 4**.

a. PFN-Style Tabular Backbone (TabPFN-v2 Encoder).

The representation backbone is replaced by a prior-data-fitted encoder that performs approximate Bayesian inference on small-sample tabular data (Zhu et al., 2023; Picard and Ahmed, 2024). A TabPFN-v2 encoder directly ingests the standardized event vector ${\text{x}}_{t}\in {\text{R}}^{2\text{CK}}$ containing robustly scaled statistics and an explicit missingness channel. This design yields a stable, low-variance event embedding suited for isotope-informed ternary component apportionment while preserving the physics-based closure used downstream.

**Figure 5:**
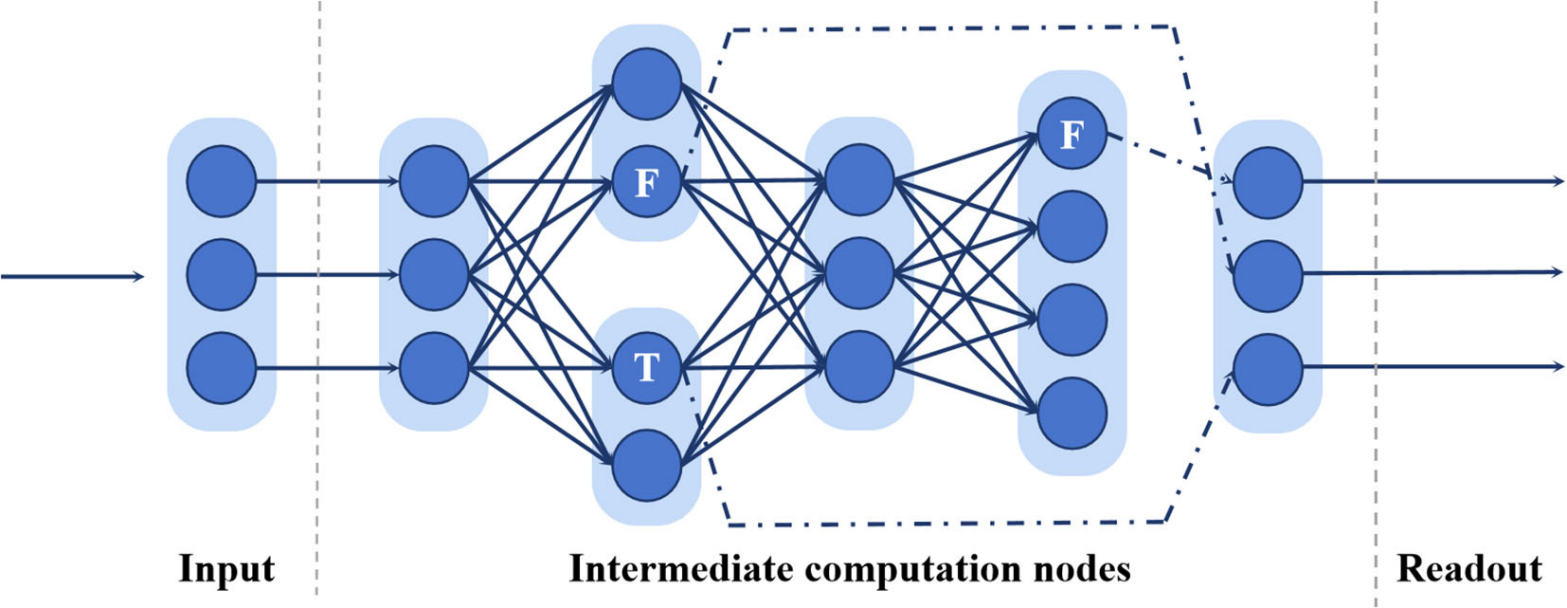
Random computational graph for synthetic TabPFN tasks. Synthetic datasets used for PFN pretraining are generated from random computational graphs. Circles denote graph nodes and directed edges define computations. Nodes marked F are feature readout positions and nodes marked T are target readout positions. Sampling many such graphs yields diverse small-sample tabular tasks that supervise the prior Θ^*^.

Let x_t_ be the input vector. The TabPFN encoder, with frozen parameters Θ^*^ pre-trained on diverse synthetic tabular tasks, produces a hidden representation. The prior parameters Θ^*^ are obtained by pretraining on millions of synthetic tabular datasets generated from random computational graphs (**Figure 5**), which enables approximate Bayesian inference at inference time without updating the encoder (Taga et al., 2025).

(6) $${\text{H}}_{\text{t}}={\text{Enc}}_{\text{PFN}}\left({\text{x}}_{\text{t}};\Theta^{*}\right).$$

A pooled summary of H_t_ is linearly projected to the backbone embedding ${\text{e}}_{\text{t}}\in {\text{R}}^{128}$,

(7) $${\text{e}}_{\text{t}}={\text{W}}_{\text{pool}}\text{pool}\left({\text{H}}_{\text{t}}\right)+{\text{b}}_{\text{pool}}.$$

During training the encoder weights remain fixed and only the projection in the above equation, the task heads, and the physics terms are optimized. The total loss keeps its original structure:

(8) $${\text{L}}_{\text{total }}={\text{ℒ}}_{\text{quantile}}\left(\theta_{\text{Q}};{\text{e}}_{\text{t}}\right)+\alpha (\tau ){\text{L}}_{\text{phys }}\left(\theta_{\text{F}};{\text{e}}_{\text{t}}\right)+{\text{ℛ}}_{\left\{\text{PFN}\right\}}$$

where θ_Q_ and θ_F_ denote the parameters of the quantile and fraction heads, and α(τ) is the epoch-dependent physical-loss weight. In this study, α(τ) followed a linear warm-up schedule: α(τ) = 0.10 + 0.90 × min(τ/50, 1), where τ denotes the training epoch. Thus, the physical-loss weight increased from 0.10 at the start of training to 1.00 over the first 50 epochs, and then remained fixed at 1.00 for the remaining training epochs. The same schedule was used in all leave-one-year-out folds. Regularization applies only to newly trained linear layers and the heads:

(9) $${\text{R}}_{\text{PFN}}=\frac{\lambda_{2}}{2}\left({\left\|{\text{W}}_{\text{pool}}\right\|}_{\text{F}}^{2}+\|\theta_{Q}{\|}_{2}^{2}+\|\theta_{\text{F}}{\|}_{2}^{2}\right)$$

The embedding e_t_ is shared, without additional gating, by two task heads exactly as before: (i) a simultaneous quantile-regression head for δD and δ^18^O (P10/P50/P90), and (ii) a softmax head for component fractions (f_tr_, f_ev_, f_adv_) with simplex closure. The shared representation tightens the link between statistical evidence and physical consistency: softmax-derived fractions and end-member isotopic values remain coupled to ground-level isotope predictions through the same unified loss as in the original design, while the PFN prior reduces variance and improves stability in small-n regimes (Yu et al., 2025b).

b. Quantile Regression and Component-Fraction Heads

Two lightweight heads operate in parallel on the shared embedding e_t_, using only δD and δ^18^O as supervision. The quantile head produces distributional estimates for each isotope at α∈{0.1,0.5,0.9}, enabling calibrated uncertainty and a central (P50) point prediction; the component-fraction head yields a simplex-constrained partition (f_tr_, f_ev_, f_adv_) that directly interfaces with the three-component physical closure.

Quantile regression head. For isotope $\text{X}\in \left\{\delta^{18}\text{O},\delta \text{D}\right\}$, the head outputs ${\text{q}}_{\alpha }^{\left(\text{X}\right)}(\text{t})$ and is trained with the pinball loss (El Hannoun et al., 2021),

(10) $${\text{L}}_{\text{quantile }}=\underset{\text{X}}{\sum }\underset{\alpha \in \{0.1,0.5,0.9\}}{\sum }\left[\alpha {\left(\delta_{\text{p,t}}^{\left(\text{X}\right)}-q_{\alpha }^{\left(\text{X}\right)}(\text{t})\right)}_{+}+(1-\alpha ){\left(q_{\alpha }^{\left(\text{X}\right)}(\text{t})-\delta_{\text{p,t}}^{\left(\text{X}\right)}\right)}_{+}\right]$$

where ${\left(\text{u}\right)}_{+}=\max(\text{u},0)$. Non-crossing is enforced by a monotone reparameterization around a shared location $\mu^{\left(\text{X}\right)}(\text{t})$,

(11) $${\text{q}}_{0.1}^{\left(\text{X}\right)}(\text{t})=\mu^{\left(\text{X}\right)}(\text{t})-\mathrm{softplus}\left(\Delta_{-}^{\left(\text{X}\right)}(\text{t})\right)$$

(12) $${\text{q}}_{0.5}^{\left(\text{X}\right)}(\text{t})=\mu^{\left(\text{X}\right)}(\text{t})$$

(13) $${\text{q}}_{0.9}^{\left(\text{X}\right)}(\text{t})={\text{μ}}^{\left(\text{X}\right)}(\text{t})+\;\mathrm{softplus}\left(\Delta_{+}^{\left(\text{X}\right)}(\text{t})\right)$$

so that ${\text{q}}_{0.1}^{\left(\text{X}\right)}\leq {\text{q}}_{0.5}^{\left(\text{X}\right)}\leq {\text{q}}_{0.9}^{\left(\text{X}\right)}$ holds by construction. The P50 output serves as the central ground-level prediction consumed by the physics module.

Component-fraction head. Fractions are produced via a temperature-scaled softmax,

(14) $${\text{f}}_{\text{t}}=\;softmax\left(\frac{{\text{W}}_{\text{f}}{\text{e}}_{\text{t}}+{\text{b}}_{\text{f}}}{\tau }\right),\;{\text{f}}_{\text{t}}={\left({\text{f}}_{\text{tr}},{\text{f}}_{\text{ev}},{\text{f}}_{\text{adv}}\right)}^{⊤},\tau >0$$

optionally regularized with a mild entropy penalty to avoid premature degeneracy. The resulting f_t_ is coupled to state-conditioned end-member isotopic values through the three-component mixing closure, providing a direct physical check on the distributional isotope predictions.

#### 2.3.3 Three-endmember closure for transpiration, surface evaporation, and advection

**Figure 6:**
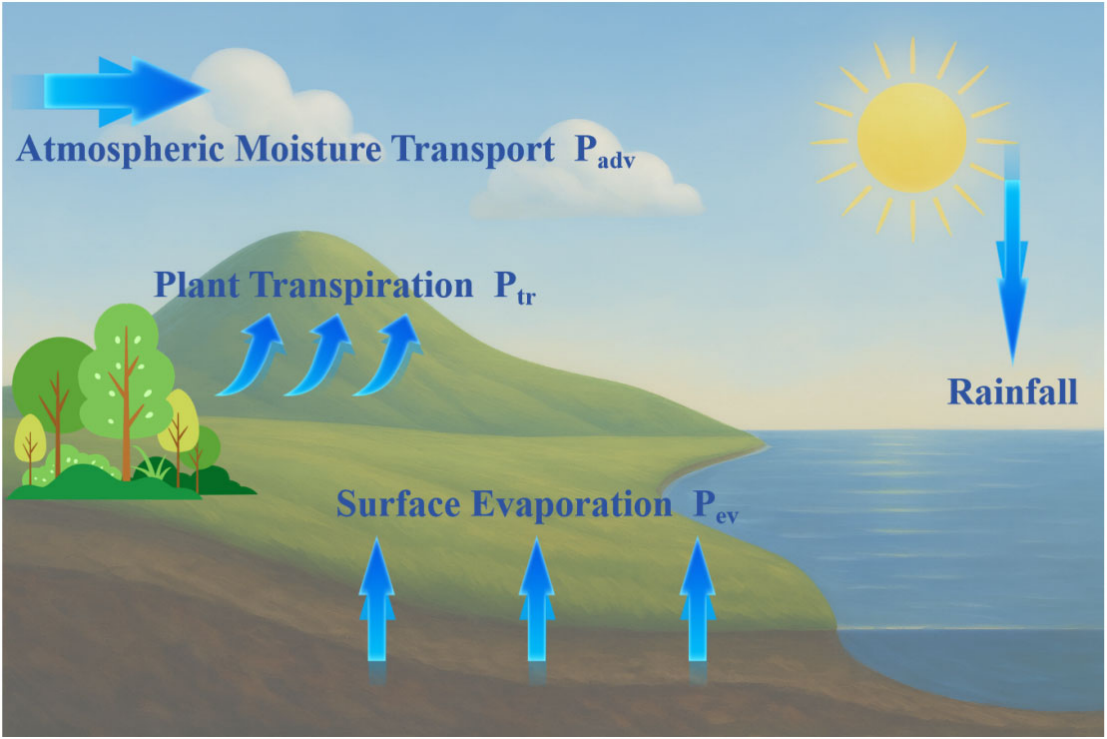
Physics-guided three-component precipitation source schematic.

To ensure that the inferred vegetation-transpiration contribution remains physically admissible, a minimal closure layer represents precipitation-forming vapor as a three-component mixture of vegetation transpiration, surface evaporation, and advected moisture (**Figure 6**) (Rine et al., 2020). The corresponding source fractions are denoted as ftr for the isotope-constrained vegetation-transpiration-associated moisture fraction, f_ev_ for the surface-evaporation fraction, and f_adv_ for the advected-moisture fraction. Here, f_tr_ should be interpreted as an ecosystem-scale source-fraction estimate rather than a direct physiological measurement of leaf transpiration. This layer links the simplex-constrained component partition with state-conditioned end-member signatures and the central isotope estimates, enforcing mass-balance closure and suppressing nonphysical source fractions. The result is a compact coupling that translates statistical evidence from isotopes, vegetation indicators, surface-energy variables, and atmospheric transport into conservation-consistent fraction fields across events (Liao and Barros, 2023; Wang and Ma, 2025).

Precipitation-forming vapor is represented as a three-component mixture of terrestrial recycling/transpiration (f_tr_), surface evaporation (f_ev_), and advection (f_adv_). The composition vector ${\text{f}}_{\text{t}}={\left({\text{f}}_{\text{tr}},{\text{f}}_{\text{ev}},{\text{f}}_{\text{adv}}\right)}^{⊤}$ is simplex-constrained,

(15) $${\text{f}}_{\text{tr}}+{\text{f}}_{\text{ev}}+{\text{f}}_{\text{adv}}=1,\;{\text{f}}_{\text{tr}},{\text{f}}_{\text{ev}},{\text{f}}_{\text{adv}}\geq 0$$

and mixes end-member signatures for each isotope $\text{X}\in \{\;\delta^{18}\text{O},\delta \text{D}\}$:

(16) $$\delta_{\text{pv,t}}^{\left(\text{X}\right)}={\text{f}}_{\text{t}}\delta_{\text{EM}}^{\left(\text{X}\right)}\left({\text{u}}_{\text{t}}\right)$$

(17) $$\delta_{\text{EM}}^{\left(E\right)}\left({\text{u}}_{\text{t}}\right)={\left[\delta_{\text{tr}}^{\left(\text{X}\right)}\left({\text{u}}_{\text{t}}\right),\delta_{\text{ev}}^{\left(\text{X}\right)}\left({\text{u}}_{\text{t}}\right),\delta_{\text{adv}}^{\left(\text{X}\right)}\left({\text{u}}_{\text{t}}\right)\right]}^{⊤}$$

Here u_t_ denotes event-level state. End-member definitions follow the standard three-component mixing model: recycling as precipitation-amount–weighted composition, evaporation in a Craig–Gordon form, and advection via a Rayleigh-type transport approximation.

To connect vapor composition to ground-level precipitation isotopes, a physically based mapping ${\text{M}}_{t}^{\left(\text{X}\right)}\left(\cdot ;{\text{u}}_{\text{t}}\right)$ is used to represent equilibrium and kinetic fractionation effects (i.e., a local-evaporation-line–type relation). Physical closure then requires the model’s central prediction ${\hat{\delta }}_{\text{p,t}}^{\left(\text{X}\right)}$ to be consistent with the three-component mixture under this mapping:

(18) $${\hat{\delta }}_{\text{p,t}}^{\left(\text{X}\right)}={\text{ℳ}}_{t}^{\left(\text{X}\right)}\left({\text{f}}_{t}^{⊤}\delta_{\text{EM}}^{\left(\text{X}\right)}\left({\text{u}}_{\text{t}}\right);{\text{u}}_{\text{t}}\right)$$

Deviations from closure are penalized through a unified physical term (Li and Zhang, 2025),

(19) $${\text{ℒ}}_{\text{phys}}=\sum_{\text{X}\in \left\{{□}^{18}\text{O,D}\right\}}\rho \left({\hat{\delta }}_{\text{p,t}}^{\left(\text{X}\right)}-{\text{ℳ}}_{t}^{\left(\text{X}\right)}\left({\text{f}}_{t}^{⊤}\delta_{\text{EM}}^{\left(\text{X}\right)}\left({\text{u}}_{\text{t}}\right);{\text{u}}_{\text{t}}\right)\right)$$

where ρ is the Huber loss with a threshold parameter h = 1.0. Specifically, ρ(e) = 0.5e² when |e| ≤ h and ρ(e) = h(|e| − 0.5h) otherwise. In implementation, the isotope residual e inside ρ was normalized by the training-fold standard deviation of the corresponding isotope to keep δ^18^O and δD on a comparable scale. In Equations 16-19, X denotes the isotope type, namely ^18^O or D; $\delta_{\text{pv,t}}^{(\text{X)}}$ denotes the isotope composition of precipitation-forming vapor for isotope X at event t; f_t_ is the simplex-constrained source-fraction vector for event t; $\delta_{\text{EM}}^{(\text{X)}}\left({\text{u}}_{\text{t}}\right)$ is the state-conditioned end-member isotope vector; u_t_ denotes the event-level environmental state derived from ERA5/MODIS predictors; $\delta_{\text{tr}}^{\left(\text{X}\right)}\left({\text{u}}_{\text{t}}\right)$, $\delta_{\text{ev}}^{\left(\text{X}\right)}\left({\text{u}}_{\text{t}}\right)$, and $\delta_{\text{adv}}^{(\text{X)}}\left({\text{u}}_{\text{t}}\right)$ denote the isotope signatures of the vegetation-transpiration, surface-evaporation, and advected-moisture endmembers, respectively; ${\text{ℳ}}_{t}^{\left(\text{X}\right)}\left(\cdot ;{\text{u}}_{\text{t}}\right)$ denotes the physically based mapping from vapor-mixture isotope composition to ground-level precipitation isotope composition; and ${\hat{\delta }}_{\text{p,t}}^{\left(\text{X}\right)}$ denotes the model-predicted central precipitation isotope value. Fractions f_t_ are produced by the simplex-constrained head through a softmax operation on the shared embedding et, while the end-member vector $\delta_{\text{EM}}^{\left(\text{X}\right)}\left({\text{u}}_{\text{t}}\right)$ is computed deterministically from the event-level state ut using standard three-component formulations. Joint training couples data-driven inference and physics:

(20) $${\text{ℒ}}_{\text{total}}={\text{ℒ}}_{\text{quantile}}+\alpha (\tau ){\text{ℒ}}_{\text{phys}}+{\text{ℛ}}_{\text{PFN}}$$

where L_total_ is the total training loss, L_quantile_ is the quantile-regression loss for δ^18^O and δD, L_phys_ is the physical closure loss defined above, and R_PFN_ is the regularization term applied to the newly trained projection and output heads. The term α(τ) denotes the epoch-dependent physical-loss weight, with τ representing the training epoch. The same linear warm-up schedule was used throughout the study: α(τ) = 0.10 + 0.90 × min(τ/50, 1). The physical-loss weight therefore increased from 0.10 to 1.00 during the first 50 training epochs and remained fixed at 1.00 thereafter, which avoided imposing the physical constraint too strongly at the beginning of training while ensuring full closure enforcement after warm-up.

Distinguishing recycled moisture f_tr_ from raindrop evaporation f_ev_ is supported by feature–mechanism alignments that act in opposite directions under common regimes. (i) Sub-cloud humidity (2-m RH and 950–800 hPa RH) and near-surface wind speed jointly modulate kinetic enrichment during below-cloud evaporation, strengthening the f_ev_ signal when air is dry and ventilation is sufficient. (ii) Boundary-layer depth and low-level wind shear control residence time and accumulation of locally recycled vapor, enhancing f_tr_ under deeper mixed layers and stronger afternoon coupling. (iii) Surface supply constraints from MODIS-based ET, NDVI, and the LST–air temperature contrast track diurnal recycling potential. Empirically, higher ET/ET_0_, deeper PBL, and stronger afternoon land–atmosphere coupling increase f_tr_, whereas low RH with shallow precipitation and weak recycling signals increase f_ev_ via enhanced kinetic enrichment. Degenerate regimes arise at high RH (> 80%), weak winds (< 3 m s^-^¹), and shallow PBL (< 0.7 km), where both mechanisms imprint similarly; in such cases we rely on the simplex prior, a mild entropy penalty on the fraction head, and the isotope-closure penalty to shrink {f_tr_, f_ev_} toward each other and avoid over-confident separation. Accordingly, we report calibrated uncertainty bands and flag low-separation cases in diagnostics.

#### 2.3.4 Evaluation of isotope reliability and source-fraction consistency

Evaluation uses a strict time-held-out design: in each fold, one calendar year is reserved for testing and all remaining years form the training set. All preprocessing statistics (winsorization bounds, medians, MADs) and all model parameters are fit on training data only and applied unchanged to the held-out year to avoid temporal leakage. The evaluation focuses on whether the framework provides reliable isotope predictions and physically consistent source fractions. Fraction diagnostics include closure residuals, simplex deviation, and association with an independent circulation-based advection index, while auxiliary isotope metrics are summarized to support the reliability of subsequent vegetation-transpiration fraction estimates. The advection index refers to a precipitation-weighted annual circulation diagnostic derived from ERA5 lower-tropospheric moisture transport and vertical motion, with larger values indicating stronger advective moisture supply and ascent conditions.

Point accuracy. Let the observed ground-level isotope be $\delta_{\text{p,t}}^{\left(\text{X}\right)}$ and the model’s central estimate ${\hat{\delta }}_{\text{p,t}}^{\left(\text{X}\right)}:={\text{q}}_{0.5}^{\left(\text{X}\right)}\left(\text{t}\right)$. Define errors ${\text{e}}_{t}^{\left(\text{X}\right)}={\hat{\delta }}_{\text{p,t}}^{\left(\text{X}\right)}-\delta_{\text{p,t}}^{\left(\text{X}\right)}$.

(21) $${\text{RMSE}}^{\left(\text{X}\right)}=\sqrt{\frac{1}{\text{n}}\sum_{t}{\left(e_{t}^{\left(X\right)}\right)}^{2}}$$

(22) $${\text{MAE}}^{\left(\text{X}\right)}=\frac{1}{\text{n}}\underset{\text{t}}{\sum }\left|{\text{e}}_{t}^{\left(\text{X}\right)}\right|$$

Distributional quality. Using predicted quantiles ${\text{q}}_{\alpha }^{\left(\text{X}\right)}(\text{t})\text{at}\;\alpha \in \{0.1,0.5,0.9\}$, the (per-event) pinball loss is:

(23) $${\text{l}}_{\alpha }^{\left(\text{X}\right)}(\text{t})=\alpha {\left(\delta_{\text{p,t}}^{\left(\text{X}\right)}-{\text{q}}_{\alpha }^{\left(\text{X}\right)}(\text{t})\right)}_{+}+(1-\alpha ){\left({\text{q}}_{\alpha }^{\left(\text{X}\right)}(\text{t})-\delta_{\text{p,t}}^{\left(\text{X}\right)}\right)}_{+}$$

reported as the mean over *α* and t. The Continuous Ranked Probability Score (CRPS), a proper scoring rule for evaluating the full predictive distribution, is computed from the quantile representation via the standard quantile-sum approximation and averaged over events.

Interval calibration and sharpness. For the nominal 80% central interval $\left[{\text{q}}_{0.1}^{\left(\text{X}\right)}(\text{t}),{\text{q}}_{0.9}^{\left(\text{X}\right)}(\text{t})\right]$:

(24) $${\text{COV}}_{80}^{\left(\text{X}\right)}=\frac{1}{\text{n}}\sum_{\text{t}}1\left\{\delta_{\text{p,t}}^{\left(\text{X}\right)}\in \left[{\text{q}}_{0.1}^{\left(\text{X}\right)}(\text{t}),{\text{q}}_{0.9}^{\left(\text{X}\right)}(\text{t})\right]\right\}$$

(25) $${\text{AIW}}_{80}^{\left(\text{X}\right)}=\frac{1}{\text{n}}\underset{\text{t}}{\sum }\left({\text{q}}_{0.9}^{\left(\text{X}\right)}(\text{t})-{\text{q}}_{0.1}^{\left(\text{X}\right)}(\text{t})\right)$$

Coverage assesses calibration against the nominal 0.80 target. Average interval width (AIW) measures the sharpness of the predictive interval, with smaller values indicating narrower uncertainty bands when coverage is maintained. We additionally report the interval score:

(26) $${\text{IS}}_{\alpha }^{\left(\text{X}\right)}=\frac{1}{\text{n}}\underset{\text{t}}{\sum }\left[\left({\text{u}}_{t}^{\left(\text{X}\right)}-{\text{l}}_{t}^{\left(\text{X}\right)}\right)+\frac{2}{\alpha }{\left({\text{l}}_{t}^{\left(\text{X}\right)}-\delta_{\text{p,t}}^{\left(\text{X}\right)}\right)}_{+}+\frac{2}{\alpha }{\left(\delta_{\text{p,t}}^{\left(\text{X}\right)}-{\text{u}}_{t}^{\left(\text{X}\right)}\right)}_{+}\right]$$

with $\alpha =0.2,{\text{l}}_{t}^{\left(\text{X}\right)}={\text{q}}_{\alpha /2}^{\left(\text{X}\right)}(\text{t}),{\text{u}}_{t}^{\left(\text{X}\right)}={\text{q}}_{1-\alpha /2}^{\left(\text{X}\right)}(\text{t})$, and the absolute calibration deviation $|{\text{COV}}_{80}^{\left(\text{X}\right)}-0.80|$.

Results are reported per fold and pooled (mean ± s.e.); model selection uses validation CRPS with early stopping, and significance is assessed with paired tests across folds. All metrics are computed only for 2018–2021 (years with isotope observations). For interpretability, we use model-agnostic KernelSHAP (1,000 background samples) applied to the standardized event-window statistics augmented with the missingness mask.

### 2.4 Computing environment

All experiments were implemented in Python 3.11 using PyTorch 2.2. Training and inference were performed on a workstation running Windows 11, equipped with an Intel Core i9–14900 CPU, 64 GB RAM, and an NVIDIA GeForce RTX 4090 GPU (24 GB VRAM).

## 3 Results

### 3.1 Isotope prediction reliability

Using the 140 event-scale precipitation-isotope samples collected during 2018–2021 and their matched ERA5/MODIS covariates described in Section 2, we first evaluated the reliability of the isotope-constrained inference. These 140 samples were used for model construction and evaluation under a leave-one-year-out design: in each fold, samples from three calendar years were used for model training, and samples from the remaining year were held out for testing. This step was used to assess whether the model could reproduce event-scale isotope variability before interpreting the derived vegetation-transpiration, surface-evaporation, and advected-moisture fractions. These isotope metrics are therefore treated as reliability diagnostics rather than as the primary ecological output of the study. All preprocessing and missing-value handling followed the procedures detailed earlier, ensuring no temporal leakage.

The model demonstrated strong point-prediction skill for both isotopes under the leave-one-year-out evaluation. Across all events, the median (P50) predictions achieved root-mean-square errors (RMSE) of 2.62‰ for δ^18^O and 19.8‰ for δD, with corresponding mean absolute errors (MAE) of 1.86‰ and 14.3‰, respectively. The coefficients of determination reached 0.61 for δ^18^O and 0.54 for δD, indicating that the satellite–reanalysis predictors captured a substantial share of event-scale isotope variability despite the limited number of precipitation samples. These error levels are consistent with the natural variability of mid-latitude precipitation isotopes and provide a stable basis for interpreting vegetationtranspiration, surface-evaporation, and advected-moisture fractions.

Probabilistic evaluation further showed well-calibrated uncertainty estimates. The nominal 80% predictive intervals attained empirical coverage of 0.83 for δ^18^O and 0.82 for δD, with average interval widths of 8.5‰ and 65.0‰, respectively. The near-nominal coverage, together with reduced CRPS values of 3.34‰ for δ^18^O and 27.8‰ for δD, indicates that the predicted distributions captured both the central tendency and event-scale dispersion without severe overconfidence.

**Figure 7:**
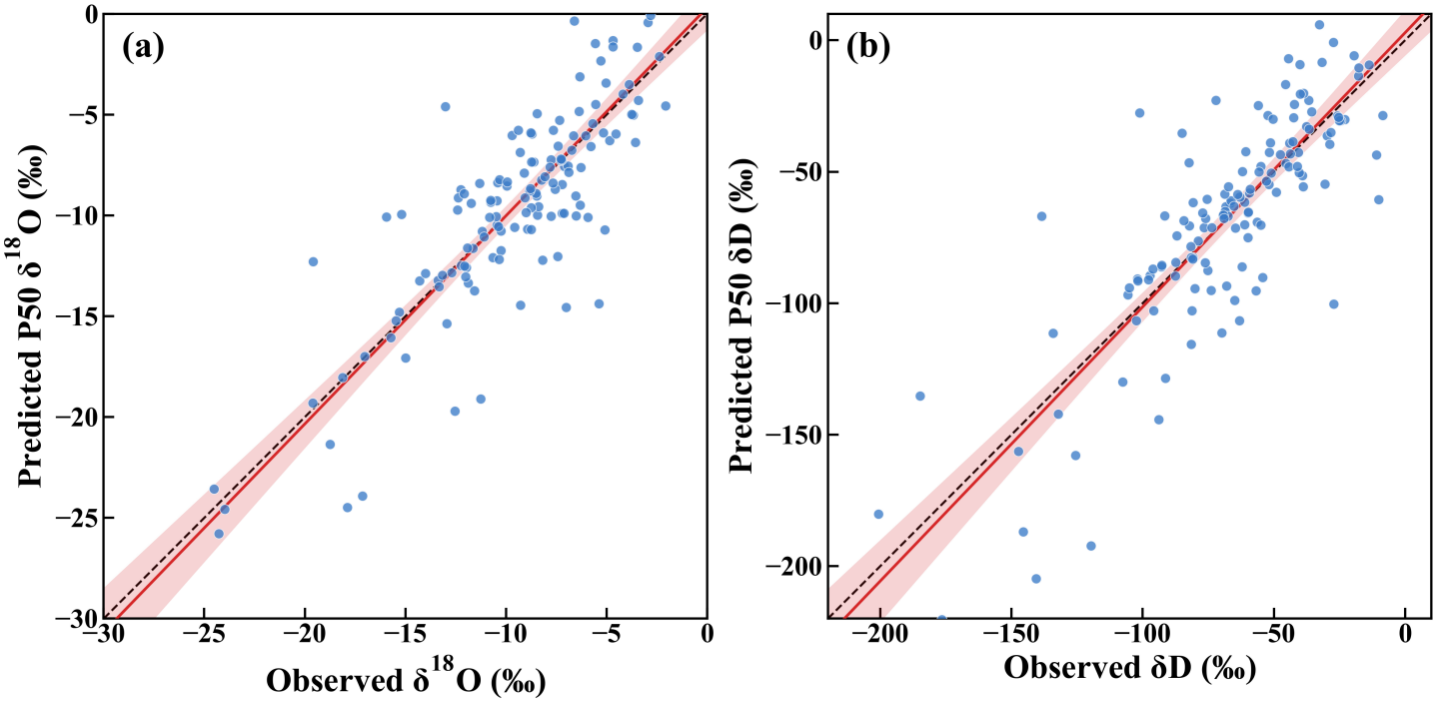
Observed–predicted scatterplots of event-scale precipitation isotopes. **(A)** δ^18^O; **(b)** δD. Each blue point represents one precipitation event. The black dashed 1:1 line indicates perfect agreement between observation and the model’s median (P50) prediction. Model performance metrics are reported in each panel and summarized as follows: δ^18^O, R² = 0.61 and RMSE = 2.62‰; δD, R² = 0.54 and RMSE = 19.8‰.

Observed–predicted scatterplots (**Figure 7**) provide a visual assessment of point-prediction skill for both isotopes.

For δ^18^O (panel a), most events cluster closely around the 1:1 line, and the residual spread is substantially narrower than in the baseline configuration. Approximately 82% of the events fall within ±3‰ of the observed value, and no systematic enrichment or depletion bias is evident. These deviations remain well within the 95% confidence envelope, confirming that the model reproduces both the mean level and the event-to-event variability of δ^18^O across the observed event record.

The δD predictions (panel b) retain a broader vertical spread, reflecting the larger natural dynamic range of δD and its stronger sensitivity to synoptic-scale advection. Nevertheless, approximately 79% of the events fall within ±20‰ of the observed values, and the fitted trend remains close to the 1:1 reference. The improved alignment indicates that the framework captures both depleted transport-dominated events and enriched local-recycling events with limited systematic bias.

**Figure 8:**
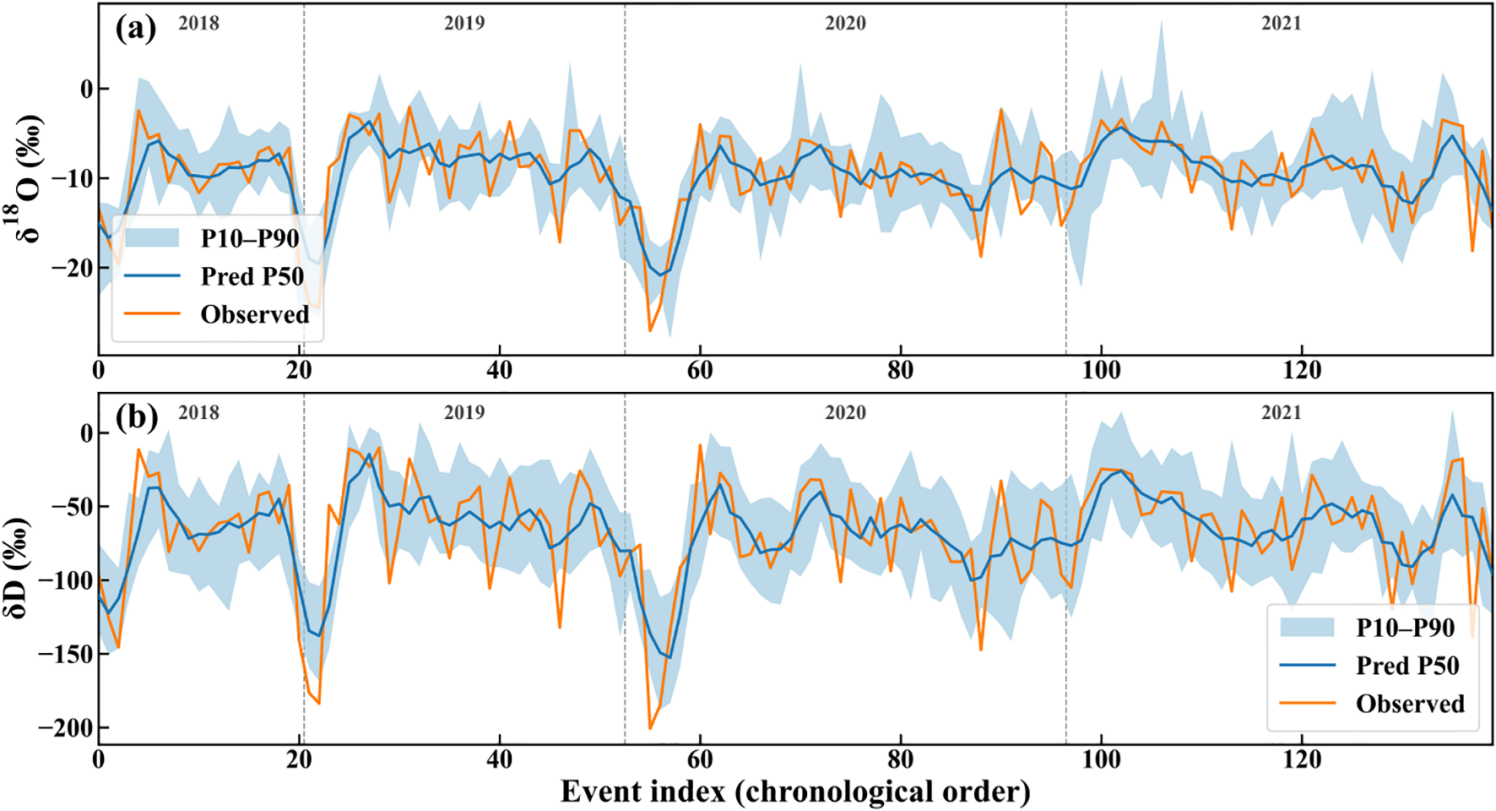
Event-scale time series of observed and predicted isotope ratios with 10th–90th percentile prediction intervals. **(A)** δ^18^O; **(B)** δD. Orange lines show observed values for 140 precipitation events ordered chronologically; blue lines denote the model median (P50) prediction; shaded bands represent the 10th–90th percentile predictive intervals (P10–P90).

**Figure 8** assesses temporal coherence of the forecasts. Across the full record, the P50 trajectory closely follows the observed sequence for both isotopes, indicating that the model captures the central tendency of event-to-event variability. Most observations fall within the P10–P90 bands, consistent with the empirical coverage of 0.83 for δ^18^O and 0.82 for δD reported in **Table 5**. Occasional exceedances of the band occur primarily at sharp isotopic excursions, where rapid transitions naturally challenge any event-scale predictor; nevertheless, deviations are short-lived and do not accumulate into systematic drift.

**Table 5:** Predictive performance of the quantile model for event-scale precipitation isotopes.

Metric	δ^18^O	δD
RMSE (‰)	2.62	19.8
MAE (‰)	1.86	14.3
R²	0.61	0.54
Pinball mean (‰)	≈0.58	≈4.86
CRPS (‰)	≈3.34	≈27.8
80% Coverage	0.83	0.82
Average Interval Width (AIW, ‰)	8.5	65

The predictive bands exhibit heteroscedastic behavior that aligns with the physics of the problem: δD shows a broader and more variable band than δ^18^O, reflecting its larger natural dynamic range and stronger sensitivity to synoptic advection. The average interval widths are 8.5‰ for δ^18^O and 65.0‰ for δD. These intervals are slightly wider than those of the lower-coverage configuration but yield near-nominal calibration, indicating that the model improves reliability without producing unrealistically narrow uncertainty bands. Taken together with the scatterplots (**Figure 7**), these time-series diagnostics demonstrate that the quantile model reproduces both the central tendency and the temporal fluctuations of event-scale precipitation isotopes while providing uncertainty bounds that are realistic—albeit slightly under-dispersed—at the nominal 80% level.

**Figure 9:**
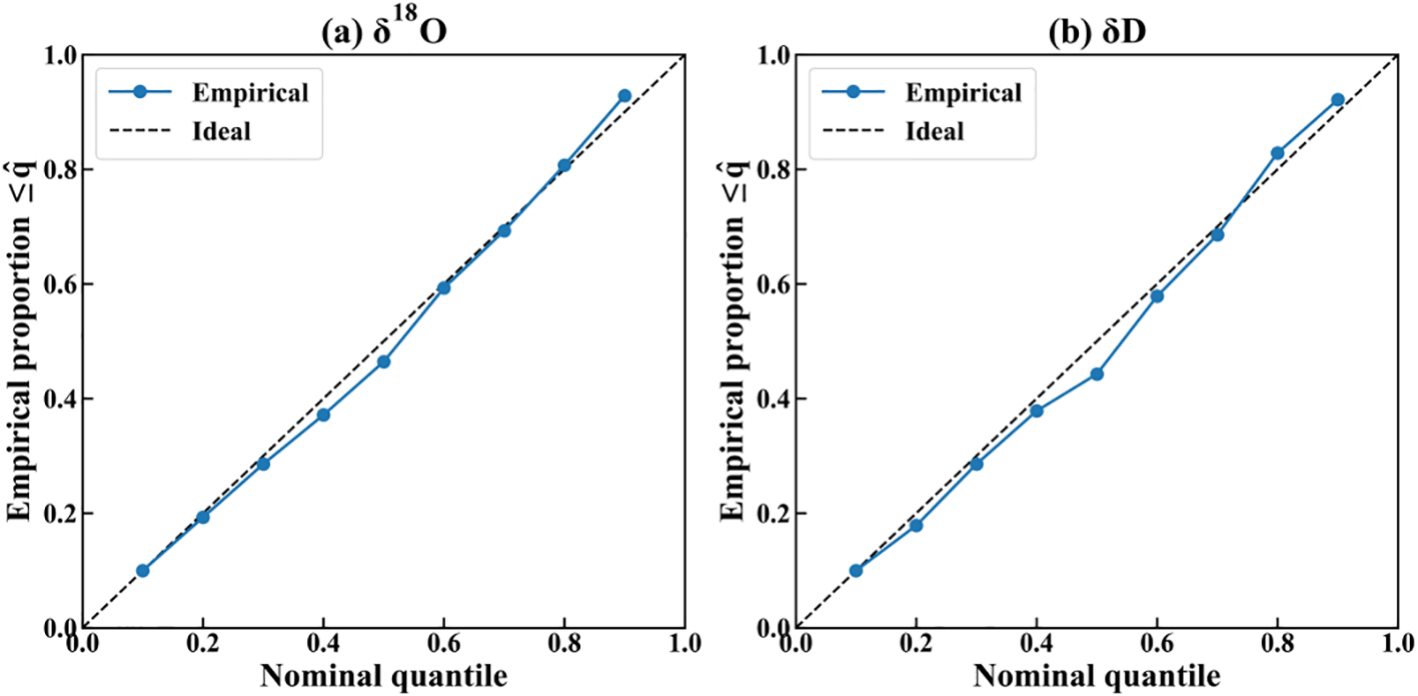
Quantile-calibration curves for event-scale isotope predictions. **(A)** δ^18^O; **(B)** δD. Blue lines with markers show the empirical proportion of observations falling below each predicted quantile; dashed lines indicate the ideal 1:1 relationship for perfect calibration.

**Figure 9** evaluates the reliability of the model’s predictive distributions. For δ^18^O (panel a), the empirical calibration curve closely follows the 1:1 reference, with only a mild underestimation of coverage at the lowest deciles (<0.3). This slight lower-tail undercoverage is mainly associated with a small number of strongly depleted or rapidly changing isotope events, for which the available ERA5/MODIS predictors and the limited event sample do not fully capture the lower-tail spread. Nevertheless, this undercoverage is limited to the lowest quantiles, and the nominal 10th–90th percentile interval still captures the main event-scale variability with near-nominal overall coverage.

For δD (panel b), the calibration curve remains close to the ideal diagonal across the full quantile range, consistent with the empirical 80% coverage of 0.82. Together with the δ^18^O coverage of 0.83, this result indicates that the quantile head provides near-nominal predictive intervals while retaining useful sharpness.

The complete leave-one-year-out procedure required approximately 31 minutes on a single RTX 4090 GPU, with a median wall-clock time of 7.8 min per fold and moderate memory demand (**Table 6**). This computational footprint remains lightweight relative to end-to-end deep-learning alternatives and is consistent with the small-sample, few-shot design.

**Table 6:** Computational footprint of the isotope-informed fraction-inversion model (leave-one-year-out CV).

Item	Value	Note
Training samples per fold	105 ± 11 events	Leave-one-year-out: remaining years after hold-out
Wall-clock time per fold	7.8 min (6.3–9.1 min)	median with range
Total CV wall-clock (4 folds)	≈31 min	single NVIDIA RTX 4090 GPU
Peak GPU utilization	~60%	from nvidia-smi profiler
Peak GPU memory	~7.8 GB	out of 24 GB
Approximately energy use	~0.37 kWh	GPU power × total runtime estimate

The cross-validation analysis shows that the model reproduces the central tendency and temporal variability of event-scale precipitation isotopes while providing reasonably calibrated but slightly under-dispersed predictive intervals. At the same time, the relatively modest explanatory power leaves open the question of whether the residual spread reflects intrinsic event-scale noise or limits in the available reanalysis and satellite predictors—a question the subsequent discussion will revisit.

### 3.2 Spatial and interannual patterns of vegetation-transpiration contributions

#### 3.2.1 Spatial distribution of plant-mediated recycling across periods

**Figure 10:**
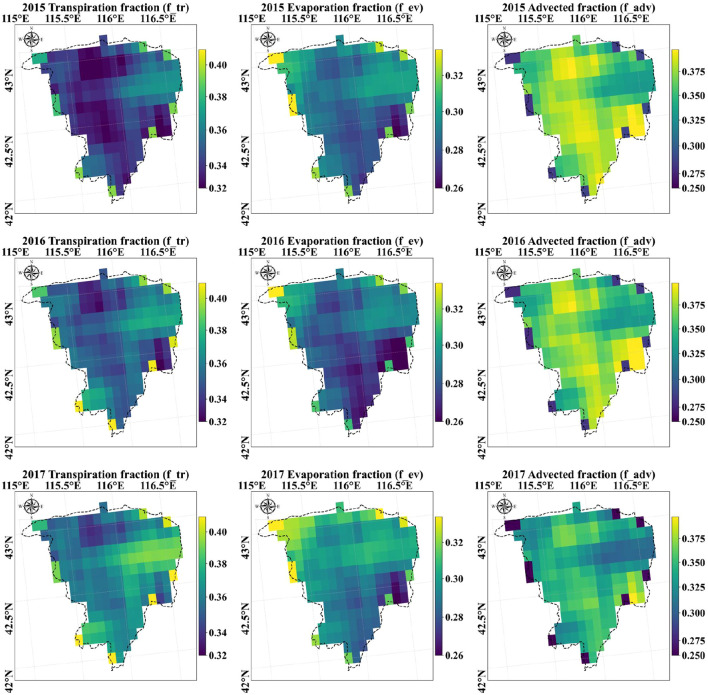
Period-mean spatial distribution of three precipitation-weighted moisture-source fractions in Zhenglan Banner during 2015–2017. Panels show the vegetation-transpiration fraction (f_tr_), surface-evaporation fraction (f_ev_), and advected-moisture fraction (f_adv_). The color scale represents dimensionless source fractions ranging from 0 to 1. The three panels use the same color scale to allow direct comparison among source fractions. Values represent precipitation-weighted annual fractions averaged over 2015–2017.

**Figure 11:**
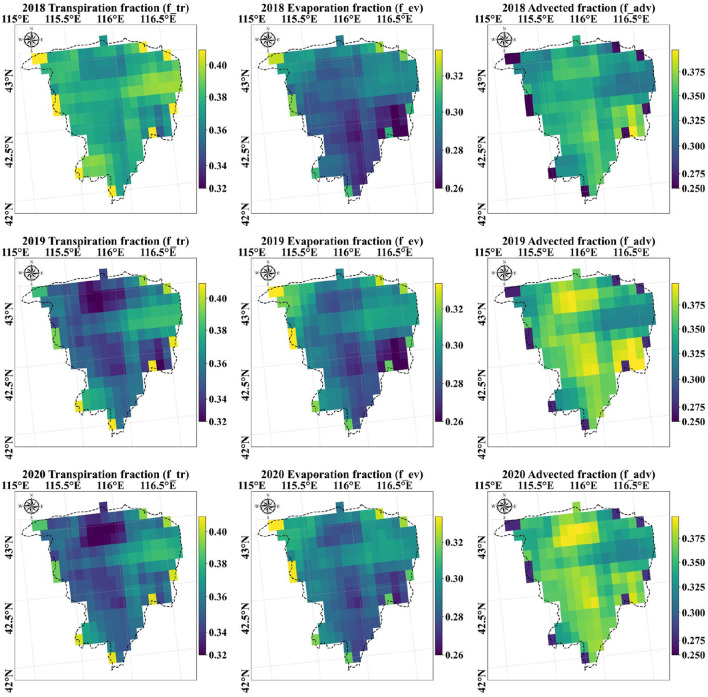
Period-mean spatial distribution of three precipitation-weighted moisture-source fractions in Zhenglan Banner during 2018–2020. Panels show the vegetation-transpiration fraction (f_tr_), surface-evaporation fraction (f_ev_), and advected-moisture fraction (f_adv_). The color scale represents dimensionless source fractions ranging from 0 to 1. The three panels use the same color scale to allow direct comparison among source fractions. Values represent precipitation-weighted annual fractions averaged over 2018–2020.

**Figure 12:**
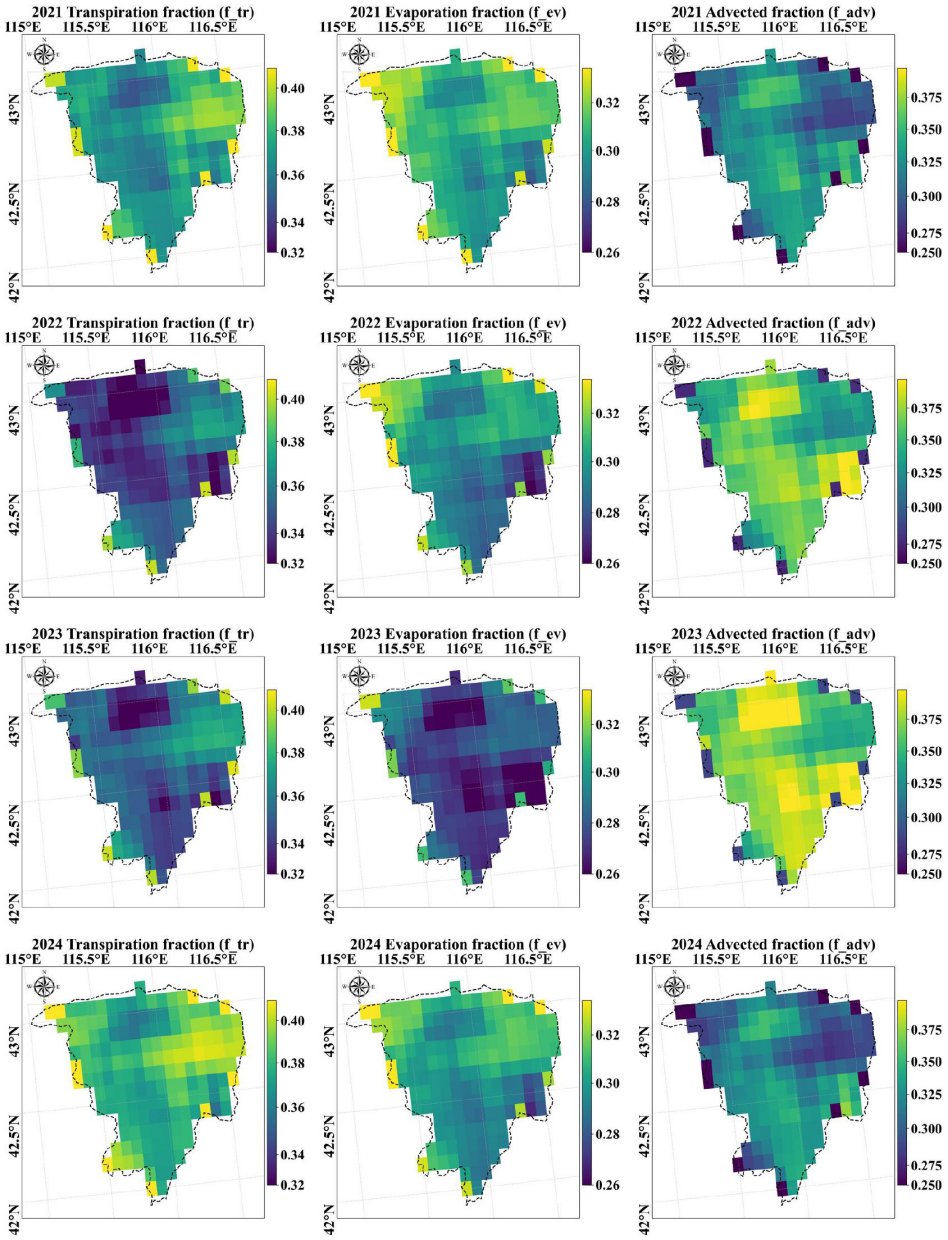
Period-mean spatial distribution of three precipitation-weighted moisture-source fractions in Zhenglan Banner during 2021–2024. Panels show the vegetation-transpiration fraction (f_tr_), surface-evaporation fraction (f_ev_), and advected-moisture fraction (f_adv_). The color scale represents dimensionless source fractions ranging from 0 to 1. The three panels use the same color scale to allow direct comparison among source fractions. Values represent precipitation-weighted annual fractions averaged over 2021–2024.

We next examined whether the inferred source fractions produced spatially coherent vegetation-transpiration patterns over Zhenglan Banner. Using the isotope-constrained framework, we retrieved precipitation-weighted annual maps of vegetation transpiration (f_tr_), surface evaporation (f_ev_), and advected moisture (f_adv_), and summarized their decadal behavior with three period means: 2015–2017, 2018–2020, and 2021–2024 (**Figures 10**–**12**). Each figure displays co-registered panels for f_tr_, f_ev_, and f_adv_ on a common color scale, enabling direct comparison across sources and periods.

A persistent backbone emerges across all periods. Transpiration forms coherent belts over vegetation-dominated terrain, while advection occupies broad lobes aligned with low-level inflow and convergence corridors; evaporation remains spatially fragmented and comparatively weak. This complementarity is required by isotopic closure—within each grid cell the three fractions sum to unity—so brightening of f_adv_ is matched by a dimming of f_tr_, and vice versa. The muted f_ev_ signal reflects both its kinetic limitation and the intermittent footprint of strong surface drying in the annual, precipitation-weighted mean.

The isotope space explains these maps mechanistically. The three endmembers occupy separated regions in the δ^18^O–δD plane: evaporation is enriched and displaced from the local meteoric water line by kinetic fractionation (low d-excess), transpiration clusters near the isotopic composition of local source water (minimal fractionation), and advected vapor projects along a Rayleigh trajectory that records upwind rainout. Period-mean shifts in the advected vertex therefore reweight f_adv_ against f_tr_ without altering the topology of the fields. Precipitation weighting further suppresses dry-month noise, ensuring that the mapped fractions reflect moisture actually delivered to the surface.

Interannual modulation rides on this backbone. The early and middle composites (2015–2017 and 2018–2020) feature expansive f_adv_ lobes and a modest contraction of transpiration belts, consistent with more depleted inflow dominating the mixing space. In contrast, the late composite (2021–2024) shows a rebound of f_tr_ and a relaxation of f_adv_ over large swaths, indicative of stronger local recycling and source-water control. Across all periods the regional means remain tightly bounded (evaporation consistently secondary), but the spatial breathing is coherent and basin-wide rather than pixel-scale.

Taken together, the three composites provide a physically consistent atlas of moisture-source partitioning: a near balance between advected inflow and transpiration that trades space from period to period, with evaporation contributing localized, low-amplitude enhancements. The agreement between the map geometry and the isotope mixing constraints gives confidence that the retrieved fractions capture genuine variability in source processes rather than artefacts of sampling or scaling.

#### 3.2.2 Interannual trade-off between vegetation-mediated recycling and advection

**Figure 13:**
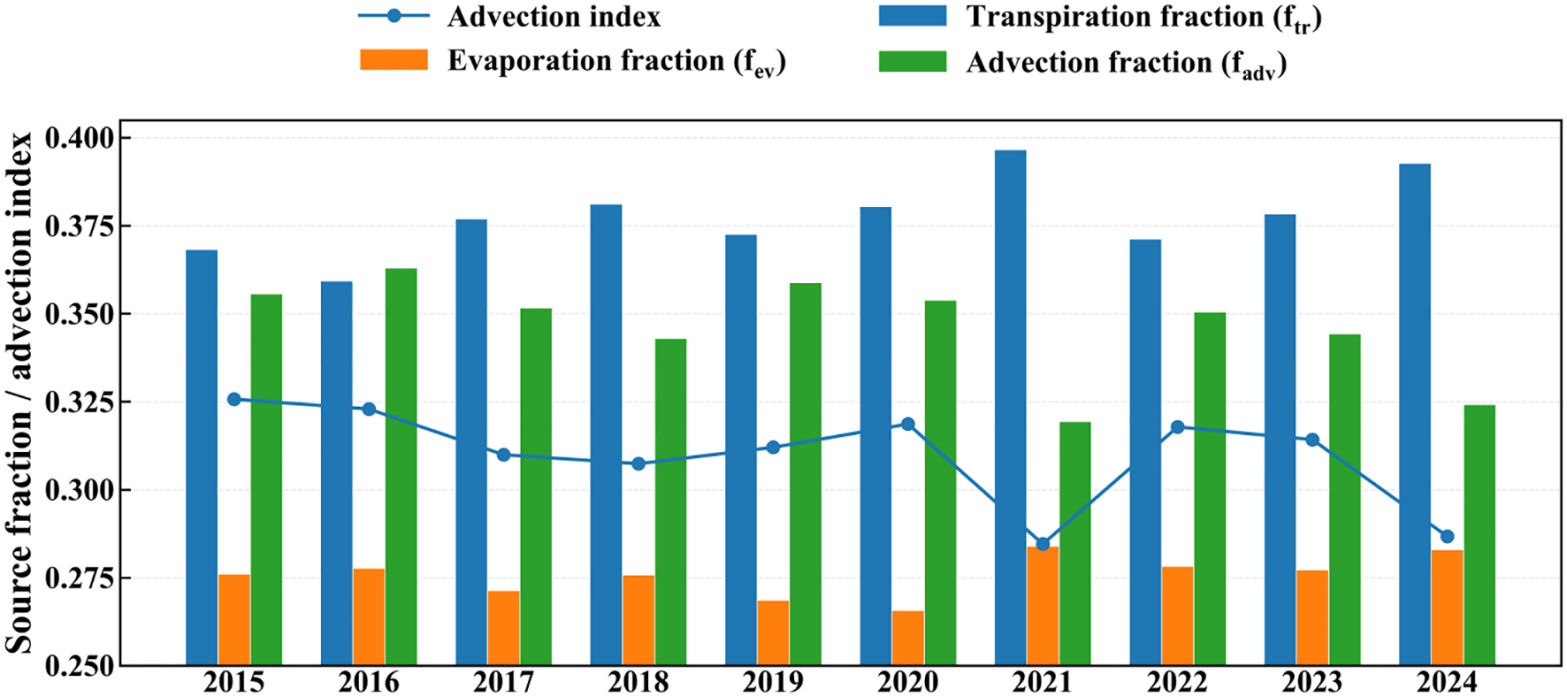
Interannual evolution of precipitation-weighted source fractions and the advection index in Zhenglan Banner from 2015 to 2024. Bars represent the annual regional mean vegetation-transpiration fraction (f_tr_), surface-evaporation fraction (f_ev_), and advected-moisture fraction (f_adv_). The line represents the precipitation-weighted advection index derived from ERA5 circulation diagnostics. The three source fractions are dimensionless and sum to one for each year, while the advection index is shown as a relative circulation indicator. Colors distinguish the plotted variables and are not used as a spatial color scale.

Here we focus on the year-to-year reorganization of the three moisture-source fractions over Zhenglan Banner from 2015 to 2024, as constrained by the isotope-informed three-endmember retrieval. The record reveals a clean, quasi one-dimensional swing along the recycling–advection axis of the mixing simplex (**Figure 13**). The annual mixtures show a clear but bounded redistribution among the three sources. The vegetation-transpiration-associated fraction, ftr, varies from 0.356 to 0.397, forming the most persistent recycled-moisture component, while fadv ranges from 0.321 to 0.365 and fev remains lower and more spatially fragmented (0.252–0.288). To avoid over-interpreting the apparent decadal tendency, we further estimated linear regression slopes and their 95% confidence intervals for the annual regional mean source fractions. The advected-moisture fraction decreased by −0.030 decade^-^¹, with a 95% confidence interval of −0.057 to −0.003 decade^-^¹. The vegetation-transpiration-associated fraction increased by +0.023 decade^-^¹, with a 95% confidence interval of +0.002 to +0.044 decade^-^¹. The surface-evaporation fraction showed only a weak increase of +0.007 decade^-^¹, with a 95% confidence interval of −0.007 to +0.021 decade^-^¹. These intervals indicate that the main interannual tendency is expressed primarily as a trade-off between advection and vegetation-associated recycling, whereas the evaporation trend is not statistically robust over the 10-year period. Given the short baseline, these results should be interpreted as evidence of a bounded decadal tendency rather than a long-term climate trend.

To test whether the retrieved transpiration fraction carried a vegetation signal rather than merely absorbing residual model error, we compared annual f_tr_ with independent MODIS vegetation and water-flux indicators. Annual f_tr_ was positively correlated with growing-season NDVI (r = +0.768), EVI (r = +0.721), and MODIS ET_daily (r = +0.794). At the spatial scale, precipitation-weighted f_tr_ also showed a positive association with mean growing-season NDVI across grid cells (r = +0.667). These relationships provide indirect consistency with the interpretation of f_tr_ as a vegetation-mediated recycling fraction rather than a purely statistical closure term, but they should not be regarded as direct validation of transpiration-derived precipitation recycling.

The timing of extrema reinforces this simplex view. Advection-dominated years—2015–2016, 2019–2020, and 2022–2023—alternate with recycling-leaning years—2021 and 2024—while 2017–2018 sit near balance. Within recycling, the internal split matters: evaporation hovers around ~0.30, providing a relatively stable kinetic endmember, whereas transpiration flexes to compensate the advection swings, thereby steering the mixture toward or away from the “vegetation” vertex of the ternary space. This geometry is borne out statistically: advection and vegetation transpiration are strongly anti-correlated (r = −0.928), confirming that the leading mode of variability trades remote inflow against vegetation-mediated recycling. Advection and evaporation are also negatively related, but less tightly (r = −0.702), whereas transpiration and evaporation show a moderate positive association (r = +0.442), consistent with their shared dependence on land-surface water and energy availability but distinct physiological and kinetic controls.

A physically independent circulation diagnostic—the precipitation-weighted advection index derived from ERA5—tracks these isotope-constrained fractions with near-monotonic covariability. The advection fraction scales closely with the precipitation-weighted advection index (r = +0.931), whereas vegetation transpiration scales oppositely (r = −0.904), indicating that stronger lower-tropospheric moisture convergence suppresses the relative contribution of local plant-mediated recycling. Evaporation shows a weaker negative association (r = −0.518), suggesting that surface evaporation remains more locally controlled and less directly paced by the regional circulation index. Years characterized by stronger lower-tropospheric moisture convergence and ascent (high index) load the mixture toward the remote-source vertex, inflating the advected share; years with weaker convergence relax toward land recycling, allowing transpiration to expand at the expense of advection (**Figure 13**). This phase-locking clarifies why the leading interannual degree of freedom is effectively one-dimensional: circulation paces the remote-versus-local balance, and the two recycling components adjust to conserve mass.

These dynamical shifts have a coherent isotopic imprint in δ^18^O–δD space. When advection increases, the annual vapor–precipitation isotope cloud is expected to translate toward the marine/remote corner, elevating the role of equilibrium transport and diminishing local kinetic imprints; when recycling strengthens, the cloud rotates toward the recycling edge, with evaporation enhancing kinetic enrichment (heavier δ^18^O at a given δD and depressed d-excess) and transpiration injecting fractionation-light recycled moisture that tempers that enrichment. The observed seesaw between advection and transpiration thus provides a mechanistic explanation for how years with similar total recycling can produce distinct isotope states depending on the internal partitioning between the two land-based sources.

Taken together, the 2015–2024 evolution presents a self-consistent narrative: circulation modulates the remote inflow; the isotope-constrained fractions respond in a nearly one-mode fashion; and the isotope systematics diagnose the internal composition of recycling. This coherence across mixing geometry, statistics, and expected isotope fingerprints supports the robustness of the retrieval and sets the stage for attributing individual years to specific circulation anomalies without invoking additional degrees of freedom.

## 4 Discussion

### 4.1 Vegetation signal in plant-mediated precipitation recycling

The main ecological implication of this study is that the inferred transpiration fraction carries a detectable vegetation signal in a semi-arid grassland, rather than acting only as a residual term in a three-component closure. Several lines of evidence support this interpretation. Spatially, f_tr_ forms coherent belts over vegetation-dominated areas, whereas f_adv_ follows inflow-related lobes and f_ev_ remains more spatially fragmented. This pattern is consistent with the expectation that plant-mediated recycling should be organized by vegetation cover, rooting access to soil or shallow groundwater, and seasonal canopy activity, rather than by atmospheric transport alone.

The independent MODIS comparisons provide a second line of support. Annual f_tr_ was positively associated with growing-season NDVI, EVI, and MODIS ET_daily, and precipitation-weighted f_tr_ also increased with mean growing-season NDVI across grid cells. These relationships do not prove leaf-level transpiration directly, but they indicate that the retrieved transpiration component covaries with vegetation greenness and water-flux indicators. Therefore, f_tr_ is best interpreted as an ecosystem-scale, isotope-constrained vegetation-transpiration-associated moisture fraction.

The interannual behavior further reinforces this interpretation. Years with weaker large-scale inflow show an expanded transpiration contribution, whereas years with stronger moisture convergence shift the mixture toward advection. This does not imply that vegetation controls regional circulation. Instead, it suggests that when advected moisture weakens, the relative contribution of land recycling becomes more visible, and the vegetation-mediated component accounts for a larger share of the precipitation-forming moisture mixture. In semi-arid grasslands, such a trade-off is ecologically meaningful because it links plant water use, canopy–atmosphere exchange, and precipitation recycling under variable hydroclimatic conditions.

At the same time, the result should be interpreted at the ecosystem scale. The present framework does not directly measure leaf transpiration, stomatal conductance, sap flow, or xylem-water isotope composition. The term f_tr_ therefore denotes an isotope-constrained, vegetation-transpiration-associated source fraction, not a direct physiological measurement of plant transpiration. This distinction is important for plant-science interpretation: the study identifies a vegetation-mediated atmospheric moisture signal that is consistent with independent vegetation indicators, while future work should connect this signal more directly to plant hydraulic and physiological observations.

### 4.2 Robustness and physical consistency of the source-partitioning framework

After establishing that the inferred f_tr_ contains a vegetation-related signal, we evaluated which framework components were necessary to maintain this interpretation under sparse event observations. We performed ablation experiments targeting five key factors: A (−Phys), removal of the three-component physical closure; B (−Mask), exclusion of the explicit missingness channel; C (−PFN), replacement of the TabPFN backbone with a conventional MLP; D (Half Data), halving of the training samples; and E (−Veg), removal of the MODIS vegetation-related predictors, including NDVI, EVI, and ET_daily. The E ablation specifically tests whether the inferred transpiration fraction is supported by vegetation and evapotranspiration information rather than being only a residual outcome of isotope closure. In D (Half Data), we randomly removed 50% of training events within each fold with a fixed seed, preserving the held-out year. **Table 7** reports the change in predictive skill relative to the full configuration (Gomez-Acebo et al., 2021).

**Table 7:** Ablation results of physics-guided TabPFN under different component combinations.

Combination	A	B	C	D	E	ΔRMSE δ^18^O	ΔRMSE δD	ΔCRPS δ^18^O	ΔCRPS δD	ΔCoverage δ^18^O	ΔCoverage δD
A	✓					1.24	8.1	1.66	10	−0.08	−0.09
B		✓				0.78	5.3	1.02	6.8	−0.05	−0.06
C			✓			0.52	3.7	0.68	4.8	−0.03	−0.04
D				✓		0.69	4.6	0.86	5.8	−0.04	−0.05
E					✓	0.93	6.4	1.18	7.8	−0.06	−0.07
A + B	✓	✓				1.78	11.4	2.34	14	−0.12	−0.13
A + D	✓			✓		1.91	12.1	2.48	15.1	−0.13	−0.14
B + C		✓	✓			1.24	8	1.6	10	−0.08	−0.09
E + D				✓	✓	1.43	9.4	1.84	11.6	−0.10	−0.10

This ablation analysis probes the logic of the framework rather than merely tallying error changes. Removing the physics-guided closure (A) severs the statistical model from the three-component mass balance it is designed to respect. Once that anchor disappears, the learned isotope fields drift: δ^18^O and δD RMSE increase by 1.24‰ and 8.1‰, respectively, and CRPS increases by 1.66‰ and 10.0‰. This degradation shows that the physics layer is not merely a *post-hoc* constraint but an active stabilizer of isotope-consistent source partitioning. This is not just a numerical penalty—it shows how strongly the TabPFN embedding relies on the closure to filter physically impossible combinations of advected vapor, surface evaporation, and transpiration. The physics layer is therefore more than a *post-hoc* check; it actively stabilizes the latent representation by enforcing physical admissibility.

Eliminating the explicit missingness mask (B) tells a complementary story. Without that binary channel, the model loses the ability to treat cloud-induced gaps as signal. Coverage and CRPS drop almost as much as in A, revealing that the absence of MODIS observations carries meteorological meaning—an implicit convection flag that, once ignored, erodes the mapping between satellite context and isotope response.

Changing the backbone from TabPFN to a plain MLP (C) or halving the training data (D) has milder but instructive effects. Both increase error by only a few tenths of a per-mil for δ^18^O, yet they arise from different mechanisms: C removes the small-sample Bayesian prior that tempers variance, while D tests data efficiency. Their similar impact suggests the PFN prior and the data volume act as parallel guards against overfitting rather than as irreplaceable physics (Bolanos-Martinez et al., 2025).

The vegetation-variable ablation (E) provides direct support for the plant-science interpretation of the framework. Removing NDVI, EVI, and ET_daily increases RMSE by 0.93‰ for δ^18^O and 6.4‰ for δD, while reducing interval coverage by 0.06–0.07. This degradation is smaller than that caused by removing the physical closure but larger than replacing the PFN backbone alone, indicating that vegetation state and evapotranspiration information make an independent contribution to constraining the transpiration-related recycling signal.

The strongest evidence for structural necessity comes from the paired ablations. When the physical closure is absent and the training set is halved (A + D), RMSE and CRPS roughly double compared with single removals, and coverage falls by more than 0.10. Here the model is doubly unmoored—physically and statistically—and the degradation is far from additive, underscoring a synergy between physical constraint and data sufficiency. A + B shows a similar compounding effect: without physics and without the meteorological cue of missingness, the network cannot separate real enrichment from sampling artifacts.

Taken together, these experiments demonstrate that the physics closure is the keystone of the architecture, the missingness mask is a quiet but powerful informer of atmospheric state, and the PFN backbone with adequate data provides the statistical discipline that allows those physical signals to be expressed. The lesson is that predictive skill emerges not from any single trick but from a negotiated balance of physical law, explicit treatment of data gaps, and a prior suited to small-sample tabular inference—a balance the full model preserves and every ablation disrupts in its own characteristic way.

A second robustness issue concerns structured missingness in MODIS inputs. MODIS covariates exhibit non-random gaps due to clouds and overpass timing. Rather than imputing or dropping these channels, we encode each predictor with a value channel (missing set to a robust center from training folds) plus a binary missingness mask. This preserves numerical stability while retaining the meteorological information carried by cloud-related outages. Consistent with the ablation results, retaining MODIS with explicit masks yields modest but systematic gains (lower CRPS and slightly improved coverage), especially during convective conditions where cloud screening is most structured; the effect is muted in non-convective cases. Importantly, the mask also prevents unwarranted confidence from naïve imputation, and together with the mixing closure it reduces non-physical source fractions when surface information is sparse.

Building on the ablation findings that highlighted the importance of the physics-guided module, we next verified that the full model’s three-component mixing layer indeed enforces a physically valid mass balance between transpiration (f_tr_), surface evaporation (f_ev_), and advected vapor (f_adv_). For each precipitation event, we reconstructed the isotope ratio implied by the predicted fractions,

(27) $$\delta_{\text{mix}}={\text{f}}_{\text{tr}}\delta_{\text{tr}}+{\text{f}}_{\text{ev}}\delta_{\text{ev}}+{\text{f}}_{\text{adv}}\delta_{\text{adv}}$$

and compared it with the observed precipitation isotopes. We also checked the simplex constraint by evaluating $\left|{\text{f}}_{\text{tr}}+{\text{f}}_{\text{ev}}+{\text{f}}_{\text{adv}}-1\right|$.

As summarized in **Table 8**, the residuals $\text{r}=\delta_{\text{obs}}-\delta_{\text{mix}}$ remain exceptionally small relative to the model’s overall predictive uncertainty. For δ^18^O, the mean residual is only 0.02‰ with an RMSE of 0.36‰, and for δD the mean residual is 0.18‰ with an RMSE of 2.72‰. These residuals are approximately 7.3 times lower than the point-prediction RMSE reported in Section 3.1 (2.62‰ and 19.8‰, respectively), indicating that most remaining prediction error arises from event-scale isotope variability rather than violation of the three-endmember closure. This tight agreement shows that the predicted end-member fractions combine to reproduce the actual precipitation isotopes with negligible bias or dispersion.

**Table 8:** Residual statistics of physics-guided closure verification.

Isotope	Mean residual (‰)	SD (‰)	RMSE (‰)	Max	Simplex deviation (mean ± max)
$\delta^{18}\text{O}$	0.02	0.36	0.36	1.05‰	0.003 ± 0.014
δD	0.18	2.71	2.72	7.8‰	0.003 ± 0.013

The simplex deviation $\left|{\text{f}}_{\text{tr}}+{\text{f}}_{\text{ev}}+{\text{f}}_{\text{adv}}-1\right|$ averages 0.003 and never exceeds 0.014, indicating that the learned fractions consistently sum to unity within numerical precision. Thus the physics layer acts not as a loose regularizer but as an active constraint that steers the solution toward conservation-consistent mixtures (Pal et al., 2022).

Taken together, these diagnostics provide quantitative evidence that the physics-guided closure operates as intended: it forces the learned fractions to obey the three-component balance and to reconstruct δ^18^O and δD with sub-per-mil oxygen and few-per-mil hydrogen residuals. Coupled with the sharp degradation observed when this module is removed in the ablation analysis above, the results establish the closure as a necessary component for translating statistical predictions into physically admissible isotope fields (Wu et al., 2023).

### 4.3 Environmental and circulation controls on moisture-source variability

**Figure 14:**
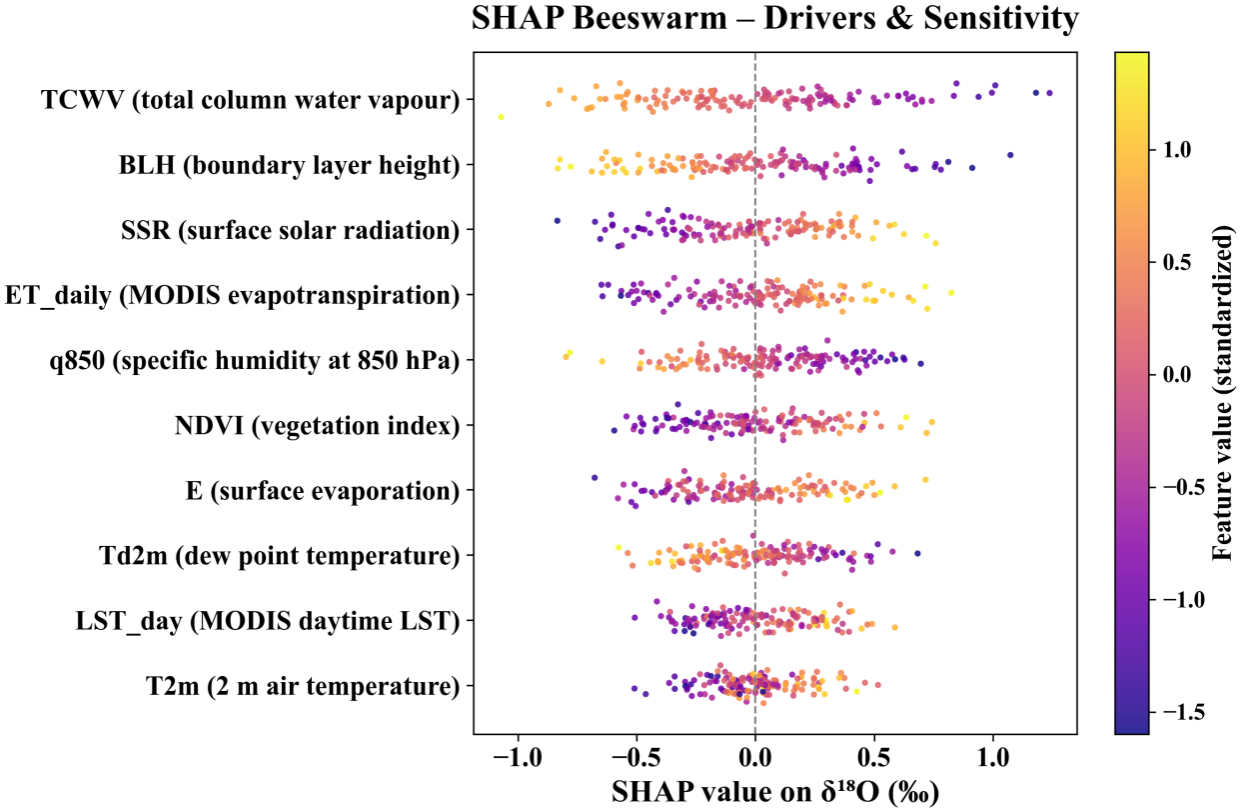
SHAP beeswarm plot of environmental drivers for event-scale δ^18^O predictions.

**Figure 15:**
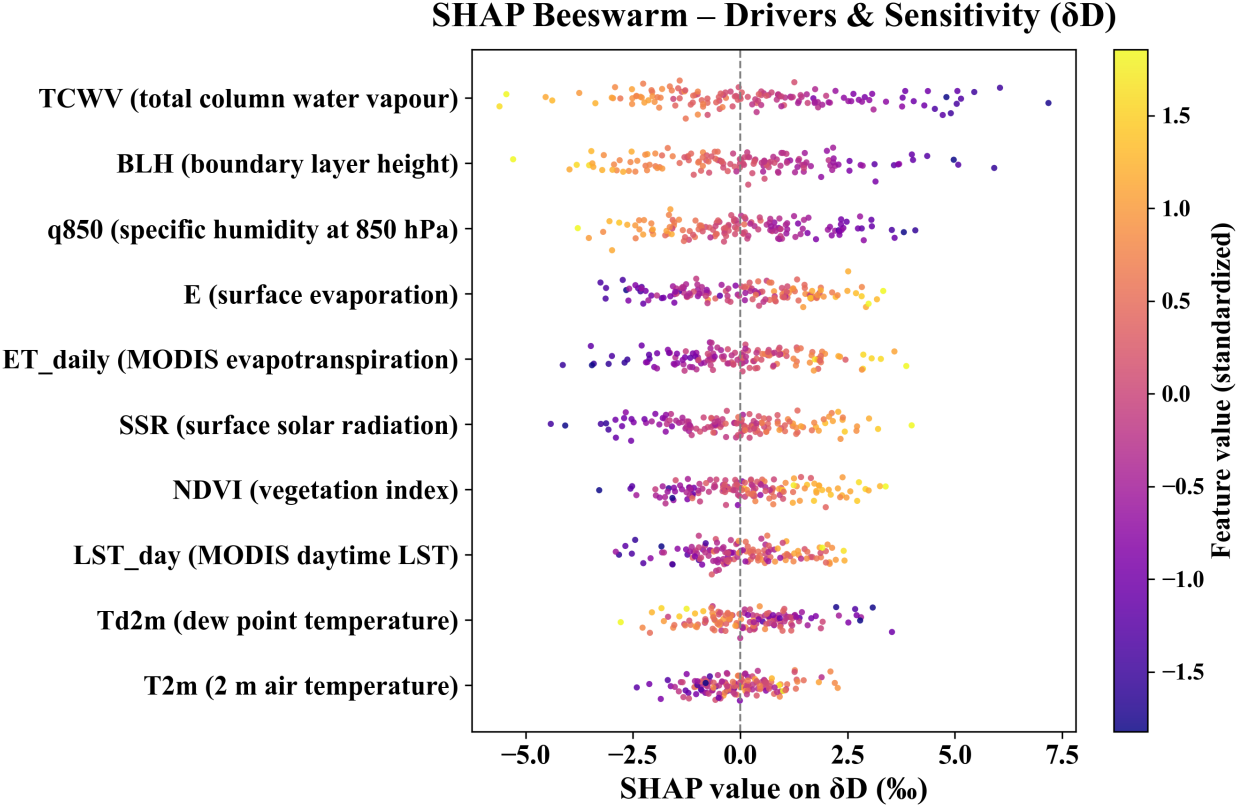
SHAP beeswarm plot of environmental drivers for event-scale δD predictions.

**Figures 14** and **15** present the SHAP analysis used to identify environmental controls on event-scale isotope variability and vegetation-related recycling signals. Each dot represents a single precipitation event, with its horizontal position denoting the SHAP contribution in per mil and its color indicating the standardized magnitude of the corresponding predictor. Across both δ^18^O and δD, the dominant controls arise from large-scale moisture transport. Total column water vapour, boundary-layer height, and mid-tropospheric specific humidity consistently rank highest in mean absolute SHAP value and show predominantly negative effects, meaning that higher values of these transport indicators drive the predicted isotope ratios downward. The pattern reflects the isotopic depletion associated with deep mixing and the advection of cooler, isotopically lighter air masses. This influence is more pronounced for δD, whose SHAP values span roughly –6.5 to +6.5‰, compared with the tighter –1.4 to +1.4‰ range observed for δ^18^O, in line with the well-known scaling of natural variability between the two isotopes (Jareemit and Srivanit, 2022; M’hamdi et al., 2024).

Secondary but coherent enrichment signals emerge from surface energy and vegetation metrics. Stronger surface solar radiation, elevated MODIS ET_daily, higher NDVI/EVI, and warmer daytime land-surface temperatures all push predictions toward heavier isotopic composition. The vegetation-related predictor group, including NDVI, EVI, and ET_daily, accounts for 32.6% of the summed mean absolute SHAP value for δ^18^O and 30.8% for δD. This contribution is smaller than that of large-scale transport variables but sufficiently large to support the interpretation that vegetation phenology and plant-mediated transpiration regulate part of the local recycling signal. Near-surface temperature and dew-point temperature exert more modest effects: warmer near-surface air slightly enriches the isotope ratios, whereas higher dew-point temperature, which reflects greater ambient humidity, tends to lower them. The magnitude of these influences again scales upward for δD while preserving the same ranking and directional consistency seen in δ^18^O.

Taken together, the paired SHAP plots demonstrate a clear physical hierarchy of controls. Large-scale atmospheric moisture supply establishes the primary isotopic baseline, while the surface energy balance and vegetation state superimpose a secondary enrichment that varies with convective activity and land–atmosphere coupling. The similarity of driver rankings across the two isotopes, despite the larger absolute amplitude of δD responses, provides strong evidence that the physics-guided TabPFN framework captures the essential mechanisms of isotope variability and maintains physically interpretable sensitivities to both transport and local-recycling processes.

Across the decade, the annual-mean mixtures occupy a remarkably thin manifold on the ternary simplex: most of the variance projects onto a single axis that trades advection against terrestrial recycling, while the evaporation share remains comparatively stable but lower than the other two components, ranging from 0.252 to 0.288 with a mean of approximately 0.27. This geometry, visible in the year-by-year barycentric means (**Figure 13**), implies a dominant mode of control rather than three independent degrees of freedom. In practice, the annual partition toggles between “remote-inflow” years, when the mixture leans toward the advected vertex and transpiration contracts, and “recycling-favored” years, when the simplex rotates toward the land-sourced edge. The narrowness of this trajectory argues that the evaporation component, though eventwise potent, integrates to a relatively stable but secondary background at the annual scale.

The circulation imprint provides a parsimonious explanation for this one-dimensional behavior (Zhang et al., 2021). Years with enhanced lower-tropospheric moisture transport and ascent—diagnosed here by stronger moisture-flux convergence and more frequent updraft—shift the isotopic end-member for the advected branch toward depleted compositions and enlarge its weight in the mixing solution. Conversely, years with weaker convergence and deeper, more weakly capped boundary layers favor surface–atmosphere coupling, allowing transpiration to expand at the expense of remote inflow. This seesaw is not symmetrical: increases in the advected fraction tend to occur abruptly in a handful of wet months that dominate precipitation weighting, whereas rebounds in transpiration accumulate across a longer season through phenology and shallow groundwater support. The resulting hysteresis helps explain why high-advection years (e.g., 2015–2016, 2019–2020, 2022–2023) cluster and why the subsequent return toward transpiration (e.g., 2021, 2024) can be sharp.

Land-surface heterogeneity modulates, rather than overturns, this circulation-paced mode. Vegetated uplands act as “recycling amplifiers,” maintaining elevated transpiration during dry spells and thus buffering the mixture away from the advected corner when large-scale inflow relaxes. In contrast, sparsely vegetated and sandy patches host intermittent kinetic enrichment that raises the evaporation signal locally but does not greatly alter the basin-wide partition once precipitation weighting is applied. This explains the empirical combination of spatial persistence and temporal plasticity: the map topology—belts of high transpiration, fragmented evaporation patches, and inflow-aligned advection lobes—remains recognizable across periods, while the net area of those motifs expands or contracts in step with the interannual mode identified above.

The isotope geometry provides an internal diagnostic that supports this interpretation (He et al., 2018; Sun et al., 2020). In the δ^18^O–δD plane, evaporation occupies the enriched, low–d-excess sector; transpiration clusters near source-water composition; and advected vapour traces a Rayleigh-like line toward depleted values. The year-to-year reweighting inferred from the fractions is therefore expected to co-translate the event cloud toward the marine/remote corner in advection-dominated years and to rotate it toward the recycling edge in transpiration-favored years. Because our fraction maps are precipitation-weighted, these isotope shifts are driven chiefly by a limited set of moisture-yielding months, which further concentrates the signal along the single leading axis seen in the ternary means.

Two broader implications follow. First, predictability: if the dominant degree of freedom is set by a handful of circulation descriptors (moisture-flux convergence, vertical velocity, column humidity), then early-season anomalies in those fields should offer advance information about which side of the advection–recycling axis the year will settle on, even before local phenology fully develops. Second, identifiability: the stability of the evaporation share at annual scale indicates that improvements in constraining the other two vertices—better characterization of upwind end-member composition in inflow years and tighter priors on source-water isotopes in recycling years—will yield disproportionate gains in fraction accuracy. Together, these points extend the descriptive maps by outlining a physically grounded, low-dimensional control space in which the decade’s spatiotemporal variability can be interpreted, tested, and—potentially—predicted.

### 4.4 Uncertainty, limitations, and transferability

The evidence presented here establishes internal validity for a single, well-instrumented site and a sample of 140 event-scale precipitation observations. Leave-one-year-out tests show only modest interannual variation in RMSE and CRPS, indicating temporal robustness within this local record (Kang et al., 2021). Those statistics, however, speak solely to the domain actually observed: they do not directly adjudicate performance under different source-moisture isotopic regimes, distinct seasonality, or alternative satellite/reanalysis characteristics. This point is especially relevant for the out-of-time reconstruction years, namely 2015–2017 and 2022–2024, for which no additional isotope observations were available. Therefore, the maps and annual fractions for these years should be interpreted as conditional model-based reconstructions constrained by ERA5/MODIS predictors and the isotope-trained three-endmember structure, rather than as independently validated isotope retrievals. Their uncertainty should be communicated more conservatively than that of the 2018–2021 isotope-observed period.

For the out-of-time years, uncertainty should be assessed and reported through three complementary checks. First, the ERA5/MODIS predictor ranges for 2015–2017 and 2022–2024 should be compared with those of the 2018–2021 training period to identify covariate-shift conditions. Years or grid cells falling outside the training predictor envelope should be flagged as lower-confidence reconstructions. Second, the P10–P90 isotope prediction intervals and physical-closure residuals should be propagated into the interpretation of source fractions, so that reconstructed ftr, fev, and fadv are presented as conditional estimates rather than exact annual values. Third, sensitivity checks on end-member assumptions, event-window aggregation, and spatial-buffer settings should be used to determine whether the main recycling–advection pattern remains stable. In this study, we therefore treat the 2015–2017 and 2022–2024 results mainly as out-of-time pattern reconstructions and emphasize robust spatial–interannual tendencies, rather than over-interpreting individual pixel-scale values or small year-to-year differences.

A second limitation concerns possible violations of the three-endmember assumption in the local hydrological setting. In Zhenglan Banner, the shallow groundwater system may influence both vegetation water use and surface evaporation. In the present framework, groundwater-derived vapor was not treated as an independent fourth endmember because it is expected to enter the atmosphere mainly through pathways already represented by the model: plant uptake followed by transpiration, or evaporation from soil, shallow water, and wet sandy surfaces. Therefore, groundwater influence is implicitly included in the vegetation-transpiration-associated fraction or the surface-evaporation fraction, depending on the dominant pathway. However, this assumption may be less reliable in local patches with persistent groundwater exposure, wetlands, lake margins, or strong capillary rise, where groundwater evaporation could have a distinct isotope signature and behave more like an additional endmember. Such areas should therefore be interpreted with greater caution.

Condensation inputs, including dew and fog, are another potential source of uncertainty. These inputs were not represented as separate source terms in the three-endmember closure because they are likely to be episodic, nocturnal, and spatially heterogeneous relative to precipitation-forming synoptic and convective moisture. Their influence is instead partly reflected through near-surface humidity, land-surface temperature, cloud-related MODIS missingness, and surface-energy predictors. Nevertheless, dew or fog deposition could modify near-surface water isotopes and subsequent evaporation after dry nights or strong radiative cooling, thereby increasing uncertainty in the surface-evaporation fraction. Future applications should test these possible fourth-endmember effects using local vapor, dew/fog, soil-water, and groundwater isotope observations, especially in low-lying or groundwater-influenced patches.

Uncertainty first arises from data and sampling. A single site cannot span the full diversity of end-member isotope signatures or synoptic configurations that drive mixing at regional scales. Although the predictors are built from globally available ERA5 and MODIS products, these inputs inherit systematic errors from retrieval algorithms, resolution mismatch, and reanalysis physics; such biases propagate into event-window summaries and, in turn, into the inferred source fractions (Hersbach et al., 2020). The assumed isotopic composition of the three source classes is likewise an approximation and may not capture the breadth of variability in real vapor origins, a recognized source of uncertainty in isotope-based mixing analysis (Parnell et al., 2013).

A second layer of uncertainty stems from model structure and representation. The three-component mixing closure f_tr_+f_ev_+f_adv_=1 is physically motivated but idealized; it can be violated during atypical synoptic situations or when additional moisture pathways are effectively present but unmodeled. Fixed event-window aggregation provides a stable covariate frame, yet it filters sub-event variability and implicitly assumes a stationary relationship between data gaps and atmospheric state. The TabPFN backbone is advantageous in small-sample regimes, but its strong prior makes predictive intervals sensitive to covariate shift—i.e., inputs falling outside the distribution seen during training (Hollmann et al., 2025).

Our uncertainty quantification is informative but conditional. The quantile head delivers P10/P50/P90 forecasts and stable CRPS values, which indicate well-calibrated dispersion for the data actually encountered. These intervals do not, by construction, account for structural misspecification, end-member mischaracterization, or shifts in input distributions; they may thus understate error when the framework is applied to climates, land-surface conditions, or sensor suites that depart from those represented here.

These considerations define both the potential and the limits of transferability. On the one hand, the input representation relies on standardized event-window statistics, and the architecture has few trainable parameters and low computational cost—attributes that make rapid deployment in other basins feasible in principle, provided comparable inputs exist. On the other hand, predictor ranges and end-member isotope signatures are expected to vary across regions, and there is no evidence that the present parameterization will remain valid under such shifts. Any extension beyond this site should therefore include (i) prior-predictive checks on covariate ranges, (ii) an audit and, if needed, recalibration of source end-member values, and (iii) at least limited local fine-tuning and out-of-sample validation.

In sum, the physics-guided TabPFN framework demonstrates temporally stable performance within one well-characterized domain and offers a data-efficient, computationally lightweight path to broader application. Its true cross-regional skill, however, remains an open question that can only be resolved through independent testing across contrasting hydro-climatic settings and alternative remote-sensing data streams.

## 5 Conclusion

This study quantified vegetation transpiration contributions to precipitation recycling in a semi-arid grassland using an isotope-constrained source-partitioning framework. By combining event-scale precipitation isotopes with ERA5 atmospheric predictors and MODIS vegetation and surface-energy indicators, we separated precipitation-forming moisture into vegetation transpiration, surface evaporation, and advected inflow under a closed three-endmember constraint.

Across 2015–2024, vegetation transpiration formed coherent belts over vegetated areas and contributed a persistent recycled-moisture component, while advected moisture varied in opposition to f_tr_ along a recycling–advection axis. The inferred f_tr_ was positively associated with growing-season NDVI, EVI, and MODIS ET_daily, supporting its interpretation as an ecosystem-scale vegetation-mediated recycling signal rather than a residual closure term. Years with weaker large-scale inflow showed expanded transpiration contribution, whereas stronger moisture convergence shifted the mixture toward advection.

The auxiliary isotope prediction and closure diagnostics support the reliability of this interpretation. The model achieved RMSE values of 2.62‰ for δ^18^O and 19.8‰ for δD, with near-nominal 80% interval coverage of 0.83 and 0.82, respectively. Closure residuals were much smaller than the total predictive error, indicating that the inferred source fractions remained physically consistent.

These findings suggest that semi-arid grassland vegetation can leave a detectable signature in precipitation recycling at the ecosystem scale. However, f_tr_ should be interpreted as an isotope-constrained vegetation-transpiration-associated moisture fraction, not as a direct leaf-level transpiration measurement. Future work should combine precipitation isotopes with sap-flow, stomatal conductance, leaf or xylem-water isotopes, and local end-member calibration to more directly connect the inferred atmospheric recycling signal with plant physiological processes.

## Data Availability

The original contributions presented in the study are included in the article/supplementary material. Further inquiries can be directed to the corresponding author.

## References

[B1] Akers P. D. Kopec B. G. Mattingly K. S. Klein E. S. Causey D. Welker J. M. (2020). Baffin Bay sea ice extent and synoptic moisture transport drive water vapor isotope (δ18O, δ2H, and deuterium excess) variability in coastal northwest Greenland. Atmos. Chem. Phys. 20, 13929–13955. doi: 10.5194/acp-20-13929-2020

[B2] Barreda J. Gomez A. Puga R. Zhou K. Zhang L. (2024). “ COSCO: A sharpness-aware training framework for few-shot multivariate time series classification”, in: Proceedings of the 33rd ACM International Conference on Information and Knowledge Management, CIKM 2024, Boise, ID, New York, NY: ACM. 3622–3626. doi: 10.1145/3627673.3679891

[B3] Bolanos-Martinez D. Duran-Lopez A. Garrido J. L. Delgado-Marquez B. Bermudez-Edo M. (2025). SASD: Self-attention for small datasets-a case study in smart villages. Expert Syst. Appl. 271, 126245. doi: 10.1016/j.eswa.2024.126245 38826717

[B4] Cai Z. Li R. Wang C. Tian L. (2025). Atmospheric controls on precipitation isotopes in North China and their response to record-breaking torrential rainfall. J. Hydrol. 661, 133762. doi: 10.1016/j.jhydrol.2025.133762 38826717

[B5] Chen Z. Liu H. Zhao J. Bi T. (2025). Small-sample event identification based on adaptive second-order MDF and triplet CNNs using distribution-level synchronized measurements. IEEE Trans. Smart Grid 16, 223–235. doi: 10.1109/TSG.2024.3454698 25079929

[B6] Cheng L. Jiang Y. Ma C. Yang J. Chen J. Yuan S. . (2023). A safety monitoring method for anchor cable prestress during slope construction based on spatial clustering and a Bayesian panel vector autoregression model. IEEE Access 11, 84860–84875. doi: 10.1109/ACCESS.2023.3303329 25079929

[B7] Cui Y. Tian L. Cai Z. Wang S. (2025). Spatially inhomogeneous response of precipitation δ18O in China to ENSO cycles. NPJ Clim. Atmos. Sci. 8. doi: 10.1038/s41612-025-01057-1 37880705

[B8] El Hannoun W. El Adlouni S.-E. Zoglat A. (2021). Vine-copula-based quantile regression for cascade reservoirs management. Water 13, 964. doi: 10.3390/w13070964 30654563

[B9] Emanuelsson B. D. Baisden W. T. Bertler N. A. N. Keller E. D. Gkinis V. (2015). High-resolution continuous-flow analysis setup for water isotopic measurement from ice cores using laser spectroscopy. Atmos. Meas. Tech. 8, 2869–2883. doi: 10.5194/amt-8-2869-2015

[B10] Feng H. Wang Y. Li Z. Zhang N. Zhang Y. Gao Y. (2023). Information leakage in deep learning-based hyperspectral image classification: A survey. Remote Sens. 15, 3793. doi: 10.3390/rs15153793 30654563

[B11] Feng X. M. Zhao Y. S. (2011). Grazing intensity monitoring in Northern China steppe: Integrating CENTURY model and MODIS data. Ecol. Indic. 11, 175–182. doi: 10.1016/j.ecolind.2009.07.002 38826717

[B12] Fu H. Neil E. J. Li H. Si B. (2025). A fully coupled numerical solution of water, vapor, heat, and water stable isotope transport in soil. Water Resour. Res. 61, e2024WR037068. doi: 10.1029/2024WR037068 29143083

[B13] Ghosh R. Renganathan A. McAliley W. Steinbach M. Duffy C. Kumar V. (2024). “ Towards entity-aware conditional variational inference for heterogeneous time-series prediction: An application to hydrology”, in: Proceedings of the 2024 SIAM International Conference on Data Mining, SDM, Houston, TX, Philadelphia, PA: Society for Industrial and Applied Mathematics. 334–342.

[B14] Gomez-Acebo I. Dierssen-Sotos T. Mirones M. Perez-Gomez B. Guevara M. Amiano P. . (2021). Adequacy of early-stage breast cancer systemic adjuvant treatment to Saint Gallen-2013 statement: The MCC-Spain study. Sci. Rep. 11, 1. doi: 10.1038/s41598-021-84825-2 33686151 PMC7970883

[B15] Goursaud S. Masson-Delmotte V. Favier V. Orsi A. Werner M. (2018). Water stable isotope spatio-temporal variability in Antarctica in 1960-2013: Observations and simulations from the ECHAM5-wiso atmospheric general circulation model. Clim. Past 14, 923–946. doi: 10.5194/cp-14-923-2018

[B16] Grande E. Moreno B. K. D. Moran J. E. (2024). A tale of two storms: Inter-storm variability of stable water isotopes in a solute transport model. Hydrol. Processes 38, e15338. doi: 10.1002/hyp.15338 41531421

[B17] Guan C. Huang M. Zhang P. (2024). “ MFORT-QA: Multi-hop few-shot open rich table question answering”, in: Proceedings of the 2024 10th International Conference on Computing and Artificial Intelligence, ICCAI 2024, Bali Island, New York, NY: ACM. 434–442. doi: 10.1145/3669754.3669822

[B18] Gunasekara R. (2025). “ Wearing privacy at risk: Privacy leakages and defense strategies in encrypted traffic forwearable devices”, in: Companion Proceedings of the ACM Web Conference 2025, Sydney, NSW, New York, NY: ACM. 689–692. doi: 10.1145/3701716.3715287

[B19] He M. Qin J. Lu N. Yao L. (2023). Assessment of ERA5 near-surface air temperatures over global oceans by combining MODIS sea surface temperature products and in-situ observations. IEEE J. Sel. Top. Appl. Earth Obs. Remote Sens. 16, 8442–8455. doi: 10.1109/JSTARS.2023.3312810 25079929

[B20] He S. Goodkin N. F. Kurita N. Wang X. Rubin C. M. (2018). Stable isotopes of precipitation during tropical Sumatra squalls in Singapore. J. Geophysical Research-Atmospheres 123, 3812–3829. doi: 10.1002/2017JD027829 41531421

[B21] Hersbach H. Bell B. Berrisford P. Hirahara S. Horányi A. Muñoz-Sabater J. . (2020). The ERA5 global reanalysis. Q. J. R. Meteorolog. Soc 146, 1999–2049. doi: 10.1002/qj.3803 41531421

[B22] Heydarizad M. Raeisi E. Sori R. Gimeno L. (2019). An overview of the atmospheric moisture transport effect on stable isotopes (δ18O, δ 2H) and D excess contents of precipitation in Iran. Theor. Appl. Climatol. 138, 47–63. doi: 10.1007/s00704-019-02798-9 30311153

[B23] Hollmann N. Müller S. Purucker L. Krishnakumar A. Körfer M. Hoo S. B. . (2025). Accurate predictions on small data with a tabular foundation model. Nature 637, 319–326. doi: 10.1038/s41586-024-08328-6 39780007 PMC11711098

[B24] Holtz D. Kaymakci C. Leuthe D. Wenninger S. Sauer A. (2025). A data-efficient active learning architecture for anomaly detection in industrial time series data. Flexible Serv. Manuf. J. doi: 10.1007/s10696-024-09588-0 30311153

[B25] Hurley J. V. Galewsky J. Worden J. Noone D. (2012). A test of the advection-condensation model for subtropical water vapor using stable isotopologue observations from Mauna Loa Observatory, Hawaii. J. Geophysical Research-Atmospheres 117. doi: 10.1029/2012JD018029 29143083

[B26] Jareemit D. Srivanit M. (2022). A comparative study of cooling performance and thermal comfort under street market shades and tree canopies in tropical savanna climate. Sustainability 14, 84653. doi: 10.3390/su14084653 30654563

[B27] Jareno F. Gonzalez M. O. Tolentino M. Sierra K. (2020). Bitcoin and gold price returns: A quantile regression and NARDL analysis. Resour. Policy 67, 101666. doi: 10.1016/j.resourpol.2020.101666 38826717

[B28] Jiang H. Yang Y. Wang H. Bai Y. Bai Y. (2020). Surface diffuse solar radiation determined by reanalysis and satellite over East Asia: Evaluation and comparison. Remote Sens. 12, 1387. doi: 10.3390/rs12091387 30654563

[B29] Johnson L. R. Sharp Z. D. Galewsky J. Strong M. Van Pelt A. D. Dong F. . (2011). Hydrogen isotope correction for laser instrument measurement bias at low water vapor concentration using conventional isotope analyses: Application to measurements from Mauna Loa Observatory, Hawaii. Rapid Commun. Mass Spectrom. 25, 608–616. doi: 10.1002/rcm.4894 21290447

[B31] Kang E. Yoo C. Shin Y. Cho D. Im J. (2021). Comparative assessment of linear regression and machine learning for analyzing the spatial distribution of ground-level NO2 concentrations: A case study for Seoul, Korea. Korean J. Remote Sens. 37, 1739–1756. doi: 10.7780/kjrs.2021.37.6.1.21

[B32] Khanmohammadi S. Cruz M. G. Perrakis D. D. B. Alexander M. E. Arashpour M. (2024). Using AutoML and generative AI to predict the type of wildfire propagation in Canadian conifer forests. Ecol. Inf. 82, 102711. doi: 10.1016/j.ecoinf.2024.102711 38826717

[B33] Kilian L. (1998). Small-sample confidence intervals for impulse response functions. Rev. Econ Stat 80, 218–230. doi: 10.1162/003465398557465

[B34] Kong Y. Pu T. Wang K. Shi X. Ren Y. Zhang W. . (2023). Ambiguity in the altitude effect of precipitation isotopes for estimating groundwater recharge elevation and paleoelevation reconstruction in the leeward side of a mountain. Hydrogeol. J. 31, 1259–1270. doi: 10.1007/s10040-023-02639-0 30311153

[B35] Kurita N. Newman B. D. Araguas-Araguas L. J. Aggarwal P. (2012). Evaluation of continuous water vapor δD and δ18O measurements by off-axis integrated cavity output spectroscopy. Atmos. Meas. Tech. 5, 2069–2080. doi: 10.5194/amt-5-2069-2012

[B36] Lang Q. Zhao W. Yu W. Ma M. Xiao Y. Huang Y. . (2023). An iterative method initialized by ERA5 reanalysis data for all-sky downward surface shortwave radiation estimation over complex terrain with MODIS observations. IEEE Trans. Geosci. Remote Sens. 61. doi: 10.1109/TGRS.2023.3323033 25079929

[B37] Lei D. Li T. Zhang L. Liu Q. Li W. (2024). A novel time-delay neural grey model and its applications. Expert Syst. Appl. 238, 121673. doi: 10.1016/j.eswa.2023.121673 38826717

[B38] Leroy-Dos Santos C. Masson-Delmotte V. Casado M. Fourre E. Steen-Larsen H. C. Maturilli M. . (2020). A 4.5 year-long record of Svalbard water vapor isotopic composition documents winter air mass origin. J. Geophysical Research-Atmospheres 125, e2020JD032681. doi: 10.1029/2020JD032681 29143083

[B39] Li C. Yu L. Zhang J. Yin S. Bao Y. Han Y. . (2014). “ Based on SPI to analysis the spatial and temporal characteristics of meteorological drought in Xilinguole League1961-2011,” in Proceedings of the 6th Annual Conference of the Risk Analysis Committee of China Association for Disaster Prevention. Beijing: China Association for Disaster Prevention. pp. 501–506.

[B40] Li W. Zhang Y. (2025). A novel solution of fluid equations for radio-frequency plasmas by physics-informed neural networks with transfer learning. Phys. Fluids 37. doi: 10.1063/5.0275335 42028584

[B41] Liao M. Barros A. P. (2023). Toward optimal rainfall for flood prediction in headwater basins-orographic QPE error modeling using machine learning. Water Resour. Res. 59, 1. doi: 10.1029/2023WR034456 29143083

[B42] Lin Z. Lin X. Li W. Tian Z. Chai X. Zou D. . (2025). Rapid few-shot tabular machine learning for Φ-OTDR event classification. Opt. Express 33, 36646–36662. doi: 10.1364/OE.571235 40984423

[B44] Liu F. Chen D. Guan Z. Zhou X. Zhu J. Ye Q. . (2024a). RemoteCLIP: A vision language foundation model for remote sensing. IEEE Trans. Geosci. Remote Sens. 62. doi: 10.1109/TGRS.2024.3390838 25079929

[B46] Liu X. Li L. Qin F. Li Y. Chen J. Fang X. (2022). Ecological policies enhanced ecosystem services in the Hunshandak sandy land of China. Ecol. Indic. 144, 109450. doi: 10.1016/j.ecolind.2022.109450 38826717

[B45] Liu J.-J. Yao J.-P. Liu J.-H. Wang Z.-Y. Huang L. (2024b). Missing data imputation and classification of small sample missing time series data based on gradient penalized adversarial multi-task learning. Appl. Intell. 54, 2528–2550. doi: 10.1007/s10489-024-05314-3 30311153

[B47] Lu C. Zhang Y. Zheng Y. Wu Z. Wang Q. (2023). Precipitable water vapor fusion of MODIS and ERA5 based on convolutional neural network. GPS Solutions 27. doi: 10.1007/s10291-022-01357-6 30311153

[B49] M’hamdi O. Takacs S. Palotas G. Ilahy R. Helyes L. Pek Z. (2024). A comparative analysis of XGBoost and neural network models for predicting some tomato fruit quality traits from environmental and meteorological data. Plants-Basel 13, 746. doi: 10.3390/plants13050746 38475592 PMC10934895

[B48] Masson-Delmotte V. Hou S. Ekaykin A. Jouzel J. Aristarain A. Bernardo R. T. . (2008). A review of Antarctic surface snow isotopic composition:: Observations, atmospheric circulation, and isotopic modeling. J. Clim. 21, 3359–3387. doi: 10.1175/2007JCLI2139.1

[B50] Mingjun Z. Shengjie W. (2016). A review of precipitation isotope studies in China: Basic pattern and hydrological process. J. Geogr. Sci. 26, 921–938. doi: 10.1007/s11442-016-1307-y 30311153

[B51] Munoz-Sabater J. Dutra E. Agusti-Panareda A. Albergel C. Arduini G. Balsamo G. . (2021). ERA5-Land: a state-of-the-art global reanalysis dataset for land applications. Earth Syst. Sci. Data 13, 4349–4383. doi: 10.5194/essd-13-4349-2021

[B52] Pal D. Bundele V. Banerjee B. Jeppu Y. (2022). SPN: stable prototypical network for few-shot learning-based hyperspectral image classification. IEEE Geosci. Remote Sens. Lett. 19, 1–5. doi: 10.1109/LGRS.2021.3085522 25079929

[B53] Pang G. Meng L. Wang X. Liu F. Shu M. Ma C. . (2025). Evaluation of surface albedo products from ERA5, JRA-55, MERRA-2, and CERES-EBAF on the Tibetan Plateau: insights from in-situ observations and MODIS data. Int. J. Digital Earth 18. doi: 10.1080/17538947.2025.2547288 37339054

[B54] Parnell A. C. Phillips D. L. Bearhop S. Semmens B. X. Ward E. J. Moore J. W. . (2013). Bayesian stable isotope mixing models. Environmetrics 24, 387–399. doi: 10.1002/env.2221 41531421

[B55] Picard C. Ahmed F. (2024). Untrained and unmatched: fast and accurate zero-training classification for tabular engineering data. J. Mech. Des. 146. doi: 10.1115/1.4064811 38635229

[B56] Qin P. Sun B. Li Z. Gao Z. Li Y. Yan Z. . (2021). Estimation of grassland carrying capacity by applying high spatiotemporal remote sensing techniques in Zhenglan Banner, Inner Mongolia, China. Sustainability 13, 3123. doi: 10.3390/su13063123 30654563

[B57] Rine M. J. McLaughlin P. I. Bancroft A. M. Harrison W. B. Kuglitsch J. Caruthers A. H. . (2020). Linked Silurian carbon cycle perturbations, bursts of pinnacle reef growth, extreme sea-level oscillations, and evaporite deposition (Michigan Basin, USA). Palaeogeogr. Palaeoclimatol. Palaeoecol. 554, 109806. doi: 10.1016/j.palaeo.2020.109806 38826717

[B58] Ruiz-Villafranca S. Roldan-Gomez J. Carrillo-Mondejar J. Martinez J. L. Ganan C. H. (2025). WFE-Tab: overcoming limitations of TabPFN in IIoT-MEC environments with a weighted fusion ensemble-TabPFN model for improved IDS performance. Future Generation Comput. Systems-The Int. J. eScience 166, 107707. doi: 10.1016/j.future.2025.107707 38826717

[B59] Rundel D. Kobialka J. von Crailsheim C. Feurer M. Nagler T. Ruegamer D. (2024). “ Interpretable machine learning for TabPFN,” in Longo L, Lapuschkin S, and Seifert C (Eds.) Explainable Artificial Intelligence. xAI 2024. Communications in Computer and Information Science, Vol. 2154. Springer, Cham. doi: 10.1007/978-3-031-63797-1_23

[B60] Stante F. Ermida S. L. DaCamara C. C. Gottsche F.-M. Trigo I. F. (2023). Impact of high concentrations of Saharan dust aerosols on infrared-based land surface temperature products. IEEE J. Sel. Top. Appl. Earth Obs. Remote Sens. 16, 4064–4079. doi: 10.1109/JSTARS.2023.3263374 25079929

[B62] Sun C. Chen W. Chen Y. Cai Z. (2020). Stable isotopes of atmospheric precipitation and its environmental drivers in the Eastern Chinese Loess Plateau, China. J. Hydrol. 581, 124404. doi: 10.1016/j.jhydrol.2019.124404 38826717

[B61] Sun B. Li Z. Gao Z. Gao W. Zhang Y. Ding X. . (2018). “ NPP estimation using time-series GF-1 data in sparse vegetation area -a case study in Zhenglanqi of Innner Monglolia, China”, in: IGARSS 2018 - 2018 IEEE International Geoscience and Remote Sensing Symposium, Valencia, Piscataway, NJ: IEEE. 3971–3974.

[B63] Szanto Z. Svingor E. Futo I. Palcsu L. Molnar M. Rinyu L. (2007). A hydrochemical and isotopic case study around a near surface radioactive waste disposal. Radiochim. Acta 95, 55–65. doi: 10.1524/ract.2007.95.1.55

[B64] Taga E. O. Ildiz M. E. Oymak S. (2025). “ TimePFN: effective multivariate time series forecasting with synthetic data”, in: Thirty-Ninth AAAI Conference on Artificial Intelligence, AAAI-25, Philadelphia, PA, Washington, DC: AAAI Press. 39, 20761–20769.

[B67] Wang T. Li T.-Y. Zhang J. Wu Y. Chen C.-J. Huang R. . (2020). A climatological interpretation of precipitation δ18O across Siberia and Central Asia. Water 12, 3212. doi: 10.3390/w12082132 30654563

[B65] Wang F. Ma Z. (2025). Transmission line icing prediction based on physically guided fast-slow transformer. Energies 18, 695. doi: 10.3390/en18030695 30654563

[B66] Wang N. Tian J. Su S. Tian Q. (2023). A downscaling method based on MODIS product for hourly ERA5 reanalysis of land surface temperature. Remote Sens. 15, 4441. doi: 10.3390/rs15184441 30654563

[B68] Wei H. Wang J. Li M. Wen M. Lu Y. (2023). Assessing the applicability of mainstream global isoscapes for predicting δ18O, δ2H, and d-excess in precipitation across China. Water 15, 3181. doi: 10.3390/w15183181 30654563

[B69] Wu C. He H. Song R. Zhu X. Peng Z. Fu Q. . (2023). A hybrid deep learning model for regional O3 and NO2 concentrations prediction based on spatiotemporal dependencies in air quality monitoring network*. Environ. pollut. 320, 121075. doi: 10.1016/j.envpol.2023.121075 36641063

[B30] Wu J. Gao Z. Zhong B. Li Z. Liu Q. Ding X. . (2016). “ Vegetation fraction inversion and influence analysis of annual ephemeral plants on sandy land evaluation”, in: 2016 IEEE International Geoscience and Remote Sensing Symposium (IGARSS), Beijing, Piscataway, NJ: IEEE. 1364–1367. doi: 10.1109/IGARSS.2016.7729348

[B71] Xu X. Gou M. (2016). “ Analysis of comprehensive factor for formation causes on dust storm hazard in Zhenglan Banner”, in: 2016 International Conference on Environmental Science and Engineering (ESE 2016), 386–390.

[B70] Xu J. Liu Z. (2023). Water vapour products from ERA5, MERSI-II/FY-3D, OLCI/Sentinel-3A, OLCI/Sentinel-3B, MODIS/Aqua and MODIS/Terra in Australia: a comparison against in situ GPS water vapour data. Q. J. R. Meteorolog. Soc 149, 1435–1458. doi: 10.1002/qj.4467 41531421

[B72] YanWei X. ShiChang K. YuLan Z. YongJun Z. (2011). A method for estimating the contribution of evaporative vapor from Nam Co to local atmospheric vapor based on stable isotopes of water bodies. Chin. Sci. Bull. 56, 1511–1517. doi: 10.1007/s11434-011-4467-2 30311153

[B73] Yi R. Alateng T. (2014). “ Characteristic analysis of the temperature variation in Zhenglan Banner in recent 50 years”, in: International Conference on Energy, Environment and Materials Engineering (EEME 2014), 227–230.

[B74] Yilmaz M. (2023). Consistency of spatiotemporal variability of MODIS and ERA5-Land surface warming trends over complex topography. Environ. Sci. pollut. Res. 30, 94414–94435. doi: 10.1007/s11356-023-28983-y 37531063

[B75] Yoshimura K. Oki T. Ohte N. Kanae S. (2003). A quantitative analysis of short-term 18O variability with a Rayleigh-type isotope circulation model - art. no. 4647. J. Geophysical Research-Atmospheres 108. doi: 10.1029/2003JD003477 29143083

[B76] Yu J. Gao X. Wang T. Lu H. Li B. Zhai F. . (2025a). A feature matching-based method for few-shot multivariate time series anomaly detection with symmetric patch mask Siam Transformer. Eng. Appl. Artif. Intell. 154, 110894. doi: 10.1016/j.engappai.2025.110894 38826717

[B77] Yu Z. Yu R. Ge X. Fu J. Hu Y. Chen S. (2025b). Tabular prior-data fitted network for urban air temperature inference and high temperature risk assessment. Sustain. Cities Soc 128, 106484. doi: 10.1016/j.scs.2025.106484 38826717

[B78] Zandler H. Senftl T. Vanselow K. A. (2020). Reanalysis datasets outperform other gridded climate products in vegetation change analysis in peripheral conservation areas of Central Asia. Sci. Rep. 10, 22446. doi: 10.1038/s41598-020-79480-y 33384431 PMC7775429

[B79] Zhang J. Li Y. Yang X. Jiang R. Zhang L. (2025). RSAM-Seg: a SAM-based model with prior knowledge integration for remote sensing image semantic segmentation. Remote Sens. 17, 09588. doi: 10.3390/rs17040590 30654563

[B80] Zhang J. Yu W. Jing Z. Lewis S. Xu B. Ma Y. . (2021). Coupled effects of moisture transport pathway and convection on stable isotopes in precipitation across the East Asian Monsoon Region: implications for paleoclimate reconstruction. J. Clim. 34, 9811–9822. doi: 10.1175/JCLI-D-21-0271.1

[B81] Zhong S. Liu D. Lin L. Zhao M. Fu X. Guo F. (2020). “ A novel anomaly detection method for gas turbines using weight agnostic neural network search”, in: 2020 Asia-Pacific International Symposium on Advanced Reliability and Maintenance Modeling (APARM).

[B82] Zhou J.-L. Li T.-Y. (2018). A tentative study of the relationship between annual δ18O & δD variations of precipitation and atmospheric circulations-a case from Southwest China. Quat. Int. 479, 117–127. doi: 10.1016/j.quaint.2017.05.038 38826717

[B83] Zhu B. Shi X. Erickson N. Li M. Karypis G. Shoaran M. (2023). “ XTab: cross-table pretraining for tabular transformers”, in: International Conference on Machine Learning, Honolulu, H.I: PMLR 202.

